# Trajectory-Oriented Brain Vulnerability Framework for Cognitive Decline in Type 2 Diabetes

**DOI:** 10.3390/ijms27188342

**Published:** 2026-09-19

**Authors:** Jana Komel, Jasna Klen

**Affiliations:** 1 Diabetes Outpatient Clinic, Community Health Centre Koper, 6000 Koper, Slovenia; jana.komel@zd-koper.si; 2 Faculty of Health Sciences, University of Primorska, 6310 Izola, Slovenia; 3 Department of Abdominal Surgery, University Medical Centre Ljubljana, 1000 Ljubljana, Slovenia; 4 Faculty of Medicine, University of Ljubljana, 1000 Ljubljana, Slovenia

**Keywords:** type 2 diabetes mellitus, cognitive decline, brain vulnerability, molecular biomarkers, neuroimaging

## Abstract

Type 2 diabetes mellitus (T2DM) is associated with heterogeneous cognitive and functional trajectories rather than a single diabetes-specific encephalopathy. This structured narrative review proposes a conceptual, hypothesis-generating exposure–injury–structure–function framework linking metabolic, vascular, and frailty-related exposures with molecular and neurovascular injury, structural change, cognitive decline, and loss of independence. Its contribution is the temporal assignment of measurements, lagged testing between adjacent levels, and comparison with clinical-risk and unordered biomarker models. For example, a study-specific threshold of at least 10% improvement in held-out root-mean-square error for 24–36-month executive/processing-speed decline could indicate incremental value. Repeated failure of a specified temporal link challenges that link; failure to improve prediction rejects incremental predictive value, not biological plausibility. Molecular hypotheses focus on AGE–RAGE signalling, NLRP3–IL-1β activation, mitochondrial quality control, endothelial dysfunction, and neuroglial injury, although human timing is uncertain. Sodium–glucose cotransporter-2 (SGLT2) inhibitors and glucagon-like peptide-1 receptor agonists (GLP-1 RAs) are considered complementary therapeutic probes. Both offer systemic benefits, but neither has proven efficacy in preventing cognitive decline or direct target engagement in the human brain. Visceral adiposity, skeletal-muscle health, sarcopenia, sex/gender, kidney function, co-pathology, and reserve are treated as exposures or modifiers. This framework is intended for longitudinal research, not clinical staging or treatment selection.

## 1. Introduction

Type 2 diabetes mellitus (T2DM) is associated with modest reductions in cognitive performance and increased long-term dementia risk [1,2,3,4]. Even mild impairment may compromise medication safety, hypoglycaemia recognition, and adherence to complex treatment. The relationship is bidirectional: diabetes may contribute to cognitive decline, while declining cognition makes diabetes harder to manage. Hyperglycaemia and glycaemic variability interact with insulin resistance, hypertension, dyslipidaemia, cerebral small-vessel disease (CSVD), chronic kidney disease (CKD), inflammation, depression, sleep disturbance, and physical inactivity. Their relative contributions vary between individuals and over time. We therefore use “diabetes-associated cognitive decline” as a clinical description, not as a distinct disease entity or a synonym for Alzheimer’s disease (AD) [3,4,5,6,7,8].

The unresolved question is temporal: which exposures accumulate first, which molecular and neurovascular responses follow, and when structural brain changes, cognitive decline, and loss of independence become detectable. Existing reviews catalogue epidemiology, mechanisms, candidate biomarkers, and therapies [4,9,10,11]. Our aim is narrower and operational: to specify longitudinal links among cumulative exposure, biological injury, structure, cognition, and function, including where adiposity, muscle loss, frailty, and reserve alter those links.

The framework translates established associations into a study design that can distinguish an early exposure response from later biological injury and clinical decline. Its proposed added value is the joint requirement for temporal ordering, independently measured modifiers, and incremental prediction; none of these alone validates the sequence. For example, a fall in glycaemic variability despite stable HbA1c should precede, rather than merely accompany, change in a prespecified injury marker, and that change should predict a later executive or processing-speed outcome. Unlike staging inferred from cross-sectional abnormality distributions, this hypothesis requires repeated within-person measurements. A specified temporal link requires reproducible forward coupling after accounting for baseline burden and reverse effects. Direct, non-adjacent paths may coexist and should be estimated rather than treated as automatic refutation. Repeated failure under an adequately measured, prespecified design rejects the tested link and lag. Failure to improve held-out prediction rejects incremental predictive value for the selected measures and outcome, without by itself disproving the underlying biology. Section 11.1 specifies the worked validation design.

## 2. Review Approach and Conceptual Scope

### 2.1. Literature Search

We conducted a structured narrative review in PubMed, supplemented by backward reference screening and forward citation tracking. Searches were developed separately for each thematic domain so that the evidence base reflected the individual components and proposed links of the framework rather than a single broad disease-level search. PubMed was searched from database inception through 30 July 2026, when the final search was conducted. The core strategy combined controlled vocabulary and title/abstract terms for T2DM with terms for cognition, cognitive decline, mild cognitive impairment, and dementia. Thematic searches addressed clinical phenotypes; molecular and neurovascular mechanisms; blood biomarkers; neuroimaging; genetics, epigenetics, reserve, and sex/gender; behaviour and frailty; adiposity, body composition, and skeletal muscle; glucose-lowering therapies; and competing frameworks and validation methods. Supplementary Table S1 documents the PubMed search strategies, including MeSH terms, title/abstract terms, Boolean operators, and field tags. No PubMed language filter was applied; English-language eligibility was applied during screening. No global human-studies filter was used because experimental evidence was eligible for the mechanistic questions addressed here.

### 2.2. Eligibility Criteria

Eligible human studies included adults with T2DM, or mixed populations with separately interpretable diabetes data, and reported cognitive, functional, biomarker, imaging, or treatment outcomes relevant to a prespecified framework link. Experimental studies were retained only to clarify molecular mechanisms for which direct human-brain evidence was unavailable. Alzheimer’s disease trials not designed specifically for T2DM were included only as contextual tests of target engagement or disease modification and were not extrapolated to prevent diabetes-associated cognitive decline. We excluded type 1 or gestational diabetes-only studies; duplicate reports; studies without cognitive, brain, or prespecified mechanistic relevance; and non-peer-reviewed reports, except for consensus or reporting guidance and one explicitly identified primary congress report used solely to contextualise semaglutide exposure in cerebrospinal fluid [12]. This report was not treated as evidence of cognitive efficacy.

### 2.3. Evidence Selection and Interpretation

Selection was hierarchical rather than exhaustive: systematic reviews and meta-analyses were prioritised for breadth, randomised trials for treatment effects, prospective cohorts for temporal associations, cross-sectional studies for hypothesis generation, and experimental work for biological plausibility. Within each thematic domain, studies were prioritised according to their direct relevance to the framework link under consideration, methodological design, sample size and population relevance, longitudinality where applicable, and ability to inform temporal ordering, biological plausibility, or clinical interpretation. Screening was not undertaken independently in duplicate; potentially eligible full texts were assessed against these criteria, and selection was refined iteratively across thematic domains. “Foundational” publications were retained when they established a definition, biological construct, measurement concept, or disease model on which subsequent evidence depended. “Contextual” publications informed interpretive boundaries, target engagement, or generalisability without directly studying T2DM-associated cognitive decline. Conflicting findings were retained and weighted by design, directness, precision, consistency, and susceptibility to confounding rather than resolved by vote counting. The review was assessed against the Scale for the Assessment of Narrative Review Articles (SANRA) [13]. It was not protocol-registered or formally risk-of-bias assessed and is therefore a structured narrative review, not a systematic review; Preferred Reporting Items for Systematic Reviews and Meta-Analyses (PRISMA) reporting and meta-analysis were not undertaken. The use of AI assistance is disclosed in the Acknowledgments. Additional details are provided in Supplementary Table S2.

## 3. Clinical Expression and Heterogeneity of Cognitive Change

The clinical phenotype is the outcome that the biological model seeks to explain. Mild cognitive impairment (MCI) is common in T2DM, but pooled prevalence varies with age, education, diabetes duration, comorbidity, recruitment setting, test battery, and diagnostic threshold [1,2]. MCI denotes objective impairment with broadly preserved independence and is distinct from subjective complaints or measurable decline that remains above an MCI threshold.

Similar glycated haemoglobin (HbA1c) values can conceal differences in cumulative glycaemic exposure, variability, hypoglycaemia, vascular and kidney disease, visceral adiposity, muscle mass, physical capacity, reserve, and genetic susceptibility. A cross-sectional cognitive score cannot capture these trajectories. Longitudinal analyses should distinguish baseline performance from change and account for practice effects, dropout, intercurrent illness, treatment change, and nonlinear decline; Section 11.1 links these problems to mixed-effects and causal longitudinal methods [3,4,5].

### 3.1. Cognitive Domains and Mixed Phenotypes

At group level, T2DM is most consistently associated with slower processing speed and weaker attention, flexibility, and executive function [1,2,3,4,5]. Effects are usually small to moderate, and no profile attributes impairment to diabetes in an individual. Episodic memory is not reliably spared; age, apolipoprotein E (APOE) ε4, hippocampal atrophy, depression, sleep disturbance, and AD co-pathology can produce amnestic or multidomain patterns [3,5]. Global screening may miss domain-specific change, while sensory or motor impairment can distort timed tests; complementary measures with different task demands are therefore needed [3,4,5].

### 3.2. Timing, Diabetes Duration, and Age at Onset

Cognitive change in T2DM is usually gradual and may be nonlinear. Earlier onset and longer duration increase cumulative exposure and later dementia risk [14]. Diabetes first recognised in later life is harder to interpret because altered body composition, frailty, multimorbidity, and prodromal neurodegeneration may coexist. Models should therefore allow change points or splines and test whether age at onset, sex/gender, adiposity, sarcopenia, and frailty modify trajectory coupling rather than assuming one constant slope. Section 11.1 describes the mixed-effects, latent-change, and causal longitudinal methods proposed to test these nonlinear trajectories.

### 3.3. Cognition and Diabetes Self-Management: A Two-Way Relationship

Even mild executive dysfunction can disrupt medication use, meal planning, interpretation of glucose-sensor data, and hypoglycaemia management. Resulting medication errors, severe hypoglycaemia, or glycaemic instability may in turn worsen cognitive vulnerability. Frailty, multimorbidity, health literacy, and social support influence both sides of this relationship, while observational studies cannot establish direction or causality. In practice, cognitive vulnerability warrants review of insulin and sulfonylurea therapy, regimen complexity, glucose-monitoring support, and the possible need for caregiver involvement [15]. Experimental evidence also links severe hypoglycaemia to pericyte and blood–brain barrier dysfunction [16].

### 3.4. Choosing Cognitive Outcomes

The Mini-Mental State Examination (MMSE) gives limited coverage of executive function and processing speed, whereas the Montreal Cognitive Assessment (MoCA) remains a screening tool. Mechanistic studies should pair a global measure with prespecified domain-sensitive tests such as the Digit Symbol Substitution Test (DSST), Trail Making Test parts A and B, Digit Span Backward, and letter and category fluency, while retaining memory measures for mixed or AD-like patterns [3,4]. Interpretation should address sensory and motor demands, practice effects, alternate forms, standardised administration, and assessor blinding. Composite scores require predefined direction, standardisation, and missing-data rules; statistical change should be interpreted with instrumental function and clinically meaningful decline [3,4].

## 4. Molecular and Neurovascular Routes to Brain Vulnerability

These pathways interact and should not be read as a fixed cascade. Longitudinal testing nevertheless requires a stated temporal hypothesis for each molecular link. Exposure measures may respond over days to months; circulating inflammatory, endothelial, neuroglial, or axonal signals over approximately 3–12 months; and magnetic resonance imaging (MRI) or cognitive and functional outcomes mainly over 12–36 months. These intervals are pragmatic choices for study design, not validated human biological clocks. Analyses should test whether within-person upstream change precedes downstream change after accounting for baseline differences, reverse effects, and possible direct pathways.

### 4.1. Hyperglycaemic Flux and Oxidative Stress

Persistent hyperglycaemia increases activity in the polyol, hexosamine, protein kinase C (PKC), and advanced glycation end-product (AGE) pathways. AGE signalling through its receptor (RAGE) promotes nuclear factor kappa B (NF-κB) activation, oxidative stress, endothelial adhesion, and blood–brain barrier (BBB) vulnerability [15,17]. A testable sequence would place sustained glycaemic and carbonyl burden first; changes in soluble RAGE, adhesion molecules, or inflammatory signals next; and structural or cognitive outcomes later. A RAGE-related link would not be supported if its longitudinal readouts neither followed exposure nor predicted the prespecified downstream measure after kidney and systemic influences were considered. Experimental work further links hyperglycaemia and advanced glycation end products to changes in blood–brain barrier junctional proteins [18].

### 4.2. Glycaemic Variability, Energetic Stress, and Mitochondria

HbA1c captures average exposure but not glucose fluctuations. Glycaemic variability is associated with cognitive outcomes, although predominantly observationally [19,20,21,22]. Repeated fluctuations may produce early redox and endothelial responses and impair mitochondrial energetics; a framework-based study should therefore align continuous glucose monitoring (CGM) with pathway-informed blood sampling before later MRI and cognition, rather than infer sequence from contemporaneous measurements.

### 4.3. Insulin Signalling and Synaptic Plasticity

Brain insulin signalling supports synaptic plasticity through insulin receptor substrate–phosphoinositide 3-kinase (PI3K)–protein kinase B (Akt) pathways; reduced Akt activity may disinhibit glycogen synthase kinase-3β (GSK-3β) and favour tau phosphorylation. AMP-activated protein kinase (AMPK)–sirtuin 1 (SIRT1)–peroxisome proliferator-activated receptor-γ coactivator-1α (PGC-1α) signalling links cellular energy sensing with mitochondrial biogenesis and autophagy [17]. Peripheral insulin resistance and blood-based signalling markers do not measure brain insulin action. Their role is therefore an early, hypothesis-specific injury layer whose relevance depends on later concordant neuroimaging or cognitive change.

### 4.4. Cerebral Microvascular Disease and Blood–Brain Barrier Dysfunction

CSVD links systemic metabolic and vascular burden with cognitive slowing. Hypertension, arterial stiffness, albuminuria, kidney disease, endothelial dysfunction, and visceral adiposity may precede impaired neurovascular coupling, BBB leakage, and white-matter injury [8,23]. Urinary albumin-to-creatinine ratio, endothelial adhesion molecules, and perfusion- or BBB-sensitive imaging may serve as early indicators, whereas white-matter hyperintensity accumulation and diffusion abnormalities typically emerge later. Competing pathology and kidney function should be measured in advance because each can produce the same signals. Endothelial effects of SGLT2 inhibitors offer a related translational hypothesis [24].

### 4.5. Vascular Injury and Neuroglial Responses

Hyperglycaemia, lipid excess, adipose-tissue inflammation, mitochondrial dysfunction, and vascular injury can activate NF-κB, microglia, astrocytes, and the NLR family pyrin domain-containing 3 (NLRP3) inflammasome [25,26,27]. One plausible sequence is a metabolic or vascular perturbation followed within months by altered NLRP3-related interleukin-1β (IL-1β)/interleukin-18 (IL-18) signalling, then by neuroglial or axonal injury markers and, later, structural or cognitive change. Human evidence for this timing is limited, and a single circulating cytokine measurement cannot establish it. Reviews of microglial and astrocytic responses provide additional mechanistic context [28,29].

Small, hypothesis-driven panels are preferable to broad exploratory panels. Obesity, infection, kidney function, medication use, and systemic vascular disease must be measured because they can generate the same circulating signal [25,26,27,30]. These recurring interpretive safeguards are consolidated in Table 1 and Table 2.

### 4.6. Mitochondrial Quality Control, Autophagy, and Cell-Fate Signalling

Mitochondrial quality control connects nutrient excess and insulin resistance with calcium regulation and inflammatory signalling. Impaired mitophagy may precede neuroaxonal injury, inefficient network function, and atrophy; persistent stress may also recruit apoptosis, pyroptosis, or ferroptosis [17,26,27]. Because direct longitudinal measures from the human brain are unavailable, peripheral mitochondrial and omic signatures remain exploratory. Their value would depend on a prespecified signature changing after an intervention and preceding a downstream brain measure. For an optional exploratory substudy, a concrete candidate is oxygen-consumption-based assessment of mitochondrial respiration in freshly isolated peripheral blood mononuclear cells [31]. The protocol should specify the respiratory endpoint, cell composition and viability, processing interval, normalisation, and batch controls before analysis. This measures peripheral bioenergetics, not cerebral mitophagy; it should not be required for full-model validation or treated as a surrogate for brain mitochondrial quality control.

### 4.7. Visceral Adiposity, Skeletal Muscle, and Frailty

Visceral adiposity contributes adipokine imbalance and low-grade inflammation that may worsen insulin resistance, oxidative stress, endothelial dysfunction, and NLRP3 signalling. Sustained loss of visceral fat has been associated with less brain atrophy and better cognition, although residual confounding and selection remain possible [32]. Skeletal muscle contributes to glucose disposal and myokine signalling; sarcopenia, low strength, inactivity, and frailty may reduce metabolic and functional reserve. Sarcopenia has been associated with dementia risk in diabetes and with later cognitive impairment more broadly [33,34]. We therefore place waist circumference or imaging-derived visceral adipose tissue mainly at the exposure level, while appendicular lean mass, grip strength, gait speed, and frailty index may modify the links from exposure to injury and from injury to function. Reverse effects are also plausible because cognitive decline can reduce activity and impair nutrition.

### 4.8. Neurodegenerative Pathology and Mixed Disease

T2DM often coexists with AD pathology and may reduce compensation for vascular or neurodegenerative injury, making previously silent changes clinically apparent sooner. Amyloid-β accumulation, phosphorylated tau, neuroaxonal injury, CSVD, and reduced reserve may contribute in different proportions. T2DM is therefore treated as an amplifier of vulnerability in mixed brain disease, not as the cause of a single molecular dementia. Neither neurofilament light chain (NfL) nor glial fibrillary acidic protein (GFAP) identifies the initiating process; an amnestic profile does not confirm AD, and white-matter hyperintensities do not establish T2DM as their cause. Interpretation depends on temporally aligned change across successive trajectory levels.

### 4.9. From Molecular Pathways to Dynamic Coupling

**Figure 1:**
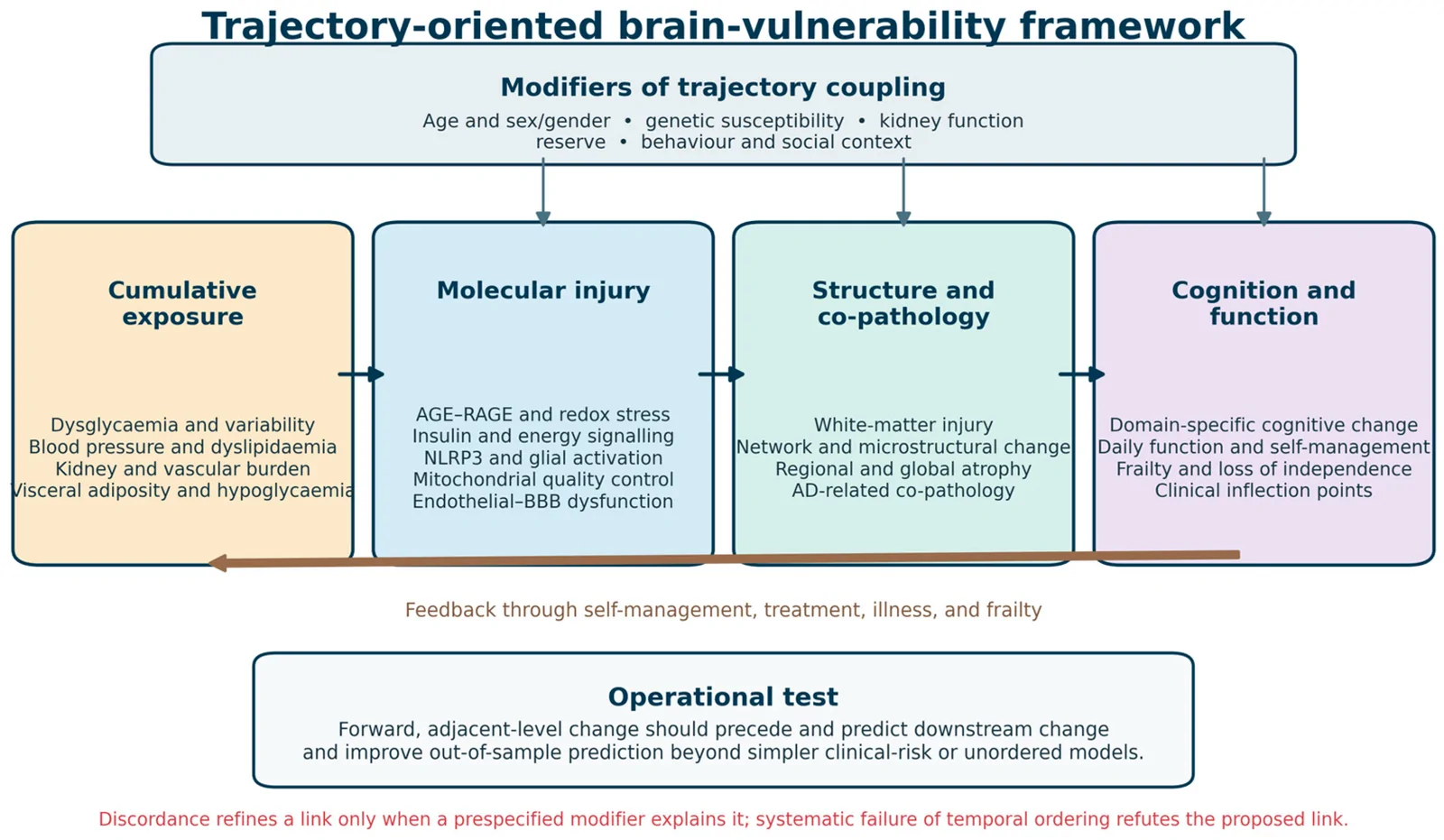
Trajectory-oriented brain-vulnerability framework. Metabolic, vascular, adiposity and hypoglycaemia exposures connect molecular injury, structure and co-pathology, and cognition and function. The upper panel identifies modifiers. Forward, reverse and direct paths are compared; direct effects may coexist with adjacent links. Temporal failure concerns the tested link and lag; predictive failure concerns incremental utility (Section 11.2). Horizontal dark arrows indicate proposed forward links between successive levels; the lower reverse arrow denotes feedback from cognition and function to upstream exposure, and downward arrows indicate possible effects of the modifiers. Abbreviations: AD, Alzheimer’s disease; AGE, advanced glycation end product; BBB, blood–brain barrier; NLRP3, NLR family pyrin domain-containing 3; RAGE, receptor for advanced glycation end products.

Table 1 sets out candidate temporal tests for the molecular network. Evidence is strongest for vascular burden, circulating injury and AD-related biomarkers, MRI, and cognitive change. By contrast, the proposed roles of NLRP3 signalling, AMPK–SIRT1–PGC-1α activity, and mitophagy rely mainly on experimental studies [17,27,35]. The intervals have not been validated. Evaluation should therefore consider temporal order, prespecified modifiers, and independent prediction together. The proposed relationships and their modifiers are illustrated in Figure 1.

Interpretive safeguards apply across the manuscript: peripheral markers do not localise cerebral activity; nonspecific markers require kidney, systemic, and co-pathology data; association is not mediation; and biological target engagement is not cognitive benefit.

## 5. Application of Molecular Biomarkers and Neuroimaging Within the Trajectory Framework

The practical task is to locate each indicator within the exposure–injury–structure–function sequence. A focused panel may combine domain-sensitive cognition, NfL and GFAP, selected AD and inflammatory biomarkers, repeated CGM, kidney and vascular phenotyping, body composition, frailty, and targeted MRI. Each measure needs one prespecified role and timescale. Table 2 consolidates maturity and limitations; CGM and adiposity are exposures, while MRI anchors structure and AD biomarkers identify co-pathology.

### 5.1. Neurofilament Light Chain and Glial Fibrillary Acidic Protein: Complementary but Nonspecific Injury Signals

NfL is sensitive to axonal injury but not its cause and may reflect CSVD, neurodegeneration, neuropathy, kidney dysfunction, or intercurrent neurological injury [36,37,38,39]. A prospective CKD cohort found strong inverse associations between estimated glomerular filtration rate (eGFR) and plasma NfL, with weaker associations for GFAP and p-tau231 [40]. GFAP reflects astrocytic responses to vascular, metabolic, and amyloid-related processes. Joint trajectories are most informative when aligned with cognition or imaging; in the Action for Health in Diabetes (Look AHEAD) study, increases in NfL and GFAP, rather than baseline levels, were associated with subsequent cognitive decline [41,42,43,44,45].

### 5.2. Alzheimer’s Disease-Related Blood Biomarkers

Plasma p-tau species and amyloid-β ratios can identify coexisting AD biology in heterogeneous T2DM populations. p-tau217 has the strongest diagnostic evidence, while p-tau231 may reflect earlier amyloid-associated change, but diabetes-specific performance is unproven [46,47]. Interpretation depends on assay, stage, age, and kidney function. Current Alzheimer’s Association guidance limits clinical use to objectively impaired patients undergoing specialist assessment and requires high assay performance before blood testing can replace cerebrospinal-fluid or amyloid-PET confirmation [48]. These criteria do not support screening asymptomatic people with diabetes.

### 5.3. Magnetic Resonance Imaging as a Structural Anchor

Conventional MRI identifies WMH, lacunes, microbleeds, infarcts, and atrophy, while diffusion MRI can detect earlier white-matter change. Their aetiology is often mixed, and longitudinal inference requires harmonised acquisition and quality control because scanner or software changes can mimic biology. Baseline MRI identifies structural vulnerability or competing lesions but not progression. Functional MRI network signatures have been linked to subsequent cognitive change in T2DM, although replication is needed [49]. Claims about WMH accumulation, diffusion change, atrophy, or neuroprotection require repeat harmonised imaging and adequate follow-up [49,50,51].

### 5.4. Aligning Continuous Glucose Monitoring, Blood Biomarkers, Imaging, and Cognition

CGM captures glucose patterns not represented by HbA1c, including time in, below, and above range; coefficient of variation; standard deviation; mean glucose; and mean amplitude of glycaemic excursions. Because these measures overlap, selection should follow a prespecified hypothesis rather than include every metric. Coordinating CGM with blood biomarkers, imaging, cognition, and everyday function permits tests of whether exposure change precedes biological injury and whether injury precedes domain-specific decline. More measurements improve inference only when their timing reflects the proposed biological sequence.

### 5.5. Biomarker Application Across Prevention, Diagnosis, and Progression

Biomarker meaning depends on clinical stage and intended use. Research use for stratification or trajectory modelling does not establish clinical utility, which additionally requires validated thresholds, incremental value, actionability, and net benefit. Treatment-related changes in NfL, GFAP, p-tau217, or MRI may support target engagement but not cognitive benefit. These are candidate intermediate endpoints, not validated surrogates; surrogacy requires trial-level evidence linking marker change to treatment effects on cognitive or functional outcomes.

### 5.6. Thresholds and Analytical Variation

No universal NfL or GFAP thresholds are validated for diabetes-associated cognitive decline. Age, kidney function, BMI, acute illness, assay characteristics, and pre-analytical handling affect concentrations. P-tau217 and amyloid assays use platform-specific algorithms that require recalibration in asymptomatic T2DM populations [36,37,38,43,44]. Reports should specify assay version, quantification limit, duplicate policy, coefficients of variation, batch allocation, storage, freeze–thaw cycles, and sample exclusions. Longitudinal change should exceed expected analytical and within-person biological variation; MRI change should surpass expected variability from scanning and segmentation, and CGM analyses require predefined minimum wear and data-completeness criteria.

## 6. Genetic, Epigenetic, and Reserve-Related Susceptibility

Similar metabolic exposure and injury do not produce the same cognitive outcome. Genetic susceptibility, epigenetic responses, cognitive and physical reserve, sex/gender, age, education, activity, mood, sleep, sarcopenia, frailty, sensory impairment, and social circumstances may alter coupling or measured performance [44,52,53,54,55,56,57,58,59]. Sex-related biology and gendered patterns of treatment, activity, education, and care access should be analysed separately where data permit; sex also affects concentrations of markers, including GFAP [44]. These factors are prespecified modifiers, not residual noise.

### 6.1. APOE ε4 as a Context-Dependent Amplifier

APOE participates in lipid transport, synaptic repair, amyloid handling, BBB integrity, and immunity. The ε4 isoform may reduce resilience to diabetes-related vascular and metabolic stress, although interaction estimates vary by cohort, age, ancestry, phenotype, and survival [60,61,62]. The relevant test is whether APOE modifies exposure-related change using prespecified interactions between exposure, genotype and time or between treatment, genotype, and time. Stratified *p*-values alone do not demonstrate interaction, and most studies are underpowered for small moderation effects [52,53].

### 6.2. Polygenic Liability and Shared Genetic Architecture

Beyond APOE, polygenic liability may represent partly separable T2DM, vascular, AD, inflammatory, lipid, and reserve pathways. Shared aetiology does not mean that a diabetes polygenic score predicts individual cognitive decline [54,55,56]. Mendelian-randomisation findings are heterogeneous and limited by instrument validity, pleiotropy, survival selection, ancestry, and the distinction between lifelong liability and treatment [56,63]. Clinical translation would require incremental discrimination and calibration beyond established risk factors. Portability across ancestry groups remains poor, so genetic enrichment is more defensible for research and triangulation than routine treatment selection [54,55,56,63].

### 6.3. Epigenetic and Transcriptional Responses to Metabolic Exposure

Metabolic memory may explain persistent effects after glycaemic exposure changes. Deoxyribonucleic acid (DNA) methylation, histone modification, chromatin accessibility, and non-coding RNAs influence inflammatory, antioxidant, endothelial, and plasticity pathways. Peripheral studies report altered brain-derived neurotrophic factor (BDNF) methylation, and experiments support SIRT1 regulation [64,65], but neither establishes human-brain effects. Blood signals are tissue- and cell-specific. Candidate and broad-omics studies therefore require prespecified tissue source, cell-composition adjustment, batch control, multiplicity correction, and replication, including attention to medication, smoking, age, and kidney function [55,56,64,65].

A recent meta-analysis found lower serum BDNF in T2DM than in healthy controls but no significant difference between participants with and without cognitive impairment [66]. Substantial heterogeneity and sensitivity to platelet release, specimen type, handling, assays, and participant characteristics support its use as an exploratory marker of neurotrophic support, not a diagnostic biomarker.

Genetic and reserve-related factors are modelled primarily as modifiers of trajectory coupling rather than independent predictors of cognitive decline.

## 7. Modifiable Context: Behaviour, Mood, Sleep, and Reserve

Behavioural and psychosocial modifiers are dynamic and potentially reversible. They alter glycaemic exposure, vascular risk, inflammation, reserve, test performance, adherence, and access to care. Their role as confounders, mediators, or effect modifiers should be specified before analysis rather than inferred from statistical associations alone [57,58,59].

### 7.1. Physical Activity, Body Composition, Frailty, and Cognitive Reserve

Physical activity may support cognition through metabolic, vascular, neurotrophic, and behavioural pathways, but reverse causality is likely when prodromal decline, neuropathy, pain, sarcopenia, or frailty reduces activity [33,34,57]. Trials should combine device-based activity with waist or visceral adiposity, appendicular lean mass where feasible, grip strength, gait speed, frailty, cognition, and everyday function. Cognitive reserve may delay the clinical expression of injury; skeletal-muscle and physical reserve may also buffer the translation of illness into disability. Their roles as exposure, mediator, or modifier must be prespecified.

### 7.2. Sleep, Circadian Disruption, Depression, and Stress

Sleep disruption, obstructive sleep apnoea, depression, and chronic stress affect metabolic regulation and cognition through hypoxia, neuroendocrine activation, endothelial dysfunction, inflammation, and reduced attention [58,59]. Because symptoms vary and respond to treatment, a baseline assessment is insufficient. These factors can confound testing, mediate treatment effects, or modify vulnerability; their causal role should be prespecified before adjustment. Screening supports phenotyping, but suspected sleep apnoea or major depression requires clinical evaluation.

### 7.3. Self-Management, Social Context, and Sensory Function

Self-management occurs within a social and sensory context. Health literacy, treatment cost, digital access, caregiver involvement, hearing, and vision influence both metabolic exposure and performance on cognitive tests [58]. Declining cognition may appear clinically as missed medication, difficulty interpreting glucose data, recurrent hypoglycaemia, missed appointments, or loss of meal-planning capacity. Such changes should trigger cognitive and functional assessment, while confirmed vulnerability should prompt regimen simplification, lower hypoglycaemia risk, and explicit allocation of treatment responsibilities. Behavioural phenotyping should be regarded as an integral component of mechanism-informed diabetes care rather than as a residual set of lifestyle covariates.

Collectively, these behavioural and reserve-related factors do not represent peripheral covariates but rather integral modifiers of trajectory coupling that may explain why comparable biological injury leads to different clinical outcomes.

## 8. Prevention and Therapeutic Translation

### 8.1. Multifactorial Risk Reduction

A prevention framework should predict that modifying established risks reduces later cognitive decline. The Systolic Blood Pressure Intervention Trial (SPRINT) lowered MCI risk with intensive blood-pressure control, and the Finnish Geriatric Intervention Study to Prevent Cognitive Impairment and Disability (FINGER) found modest benefit from multidomain management [67,68]. Because SPRINT excluded diabetes and multidomain trials cannot isolate active components, these studies support comprehensive risk reduction rather than a diabetes-specific mechanism. Priorities include safe individualised glycaemic targets, avoidance of severe hypoglycaemia, vascular risk control, smoking cessation, activity, and management of sleep, depression, and sensory impairment; treatment should be simplified when cognition compromises safety [58,67,68].

### 8.2. Glucose-Lowering Therapies as Mechanistic Probes

Glucose-lowering therapies offer a way to perturb upstream pathways, although none is established for dementia prevention. We selected SGLT2 inhibitors and GLP-1 RAs because both have established systemic efficacy, measurable effects on relevant exposures, plausible links to vascular or metabolic brain vulnerability, and enough human evidence for longitudinal comparison. SGLT2 inhibitors mainly perturb cardiorenal, haemodynamic, and substrate pathways, whereas GLP-1 RAs also affect appetite, adiposity, insulin signalling, and inflammation. Observational comparisons remain vulnerable to treatment selection, weight change, healthcare engagement, and differences in baseline risk [69,70,71,72,73,74,75,76,77,78].

### 8.3. SGLT2 Inhibitors and GLP-1 Receptor Agonists as Complementary Probes

SGLT2 inhibitors have established cardiorenal benefits and measurable systemic effects relevant to neurovascular vulnerability [69,70,71,79]. Several observational cohorts report lower dementia incidence among users, but residual confounding remains a concern [72,73,74,75,76,80,81]. Neither direct target engagement in the human brain nor cognitive neuroprotection has been demonstrated. Evidence from functional MRI, altered substrate utilisation, and cellular and animal models provides mechanistic support but does not establish cognitive protection [27,35,82,83,84,85]. A suitable trial would examine whether early changes in CGM, blood pressure, kidney function, or endothelial measures precede changes in injury markers and, subsequently, cognition or brain structure.

Human evidence for GLP-1 RAs is broader but remains heterogeneous. Small trials in obesity, prediabetes, or early T2DM reported improved memory with liraglutide, and other prospective or randomised studies examined functional near-infrared spectroscopy and olfactory-network activation [86,87,88]. The Researching Cardiovascular Events with a Weekly Incretin in Diabetes (REWIND) trial reported fewer cases of cognitive impairment with dulaglutide in an exploratory analysis; pooled trials and register analyses also suggested lower dementia incidence, although cognition was not their primary endpoint [89,90]. A REWIND biomarker substudy found no significant overall effect of dulaglutide on NfL, p-tau217, GFAP, or substantive cognitive impairment, although exploratory signals were observed in biomarker-defined high-risk subgroups [91]. A 24-month prospective non-randomised study in adults with T2DM and MCI associated semaglutide with improved MoCA and DSST scores and less progression to dementia, but its small sample, treatment selection, practice effects, and concurrent weight and glycaemic changes preclude causal inference [92]. Target-trial emulations have associated semaglutide or the class with fewer AD-related diagnoses [76,93], whereas another target-trial emulation in older adults found no clear overall difference in dementia incidence between GLP-1 RAs and dipeptidyl peptidase-4 (DPP-4) inhibitors [94]. Outcome misclassification, treatment selection, weight-loss mediation, and short follow-up limit these observational findings. The Evaluating Liraglutide in Alzheimer’s Disease (ELAD) trial enrolled people with established AD without diabetes [95].

The phase 3 EVOKE and EVOKE+ trials did not significantly slow clinical progression at week 104 in 3808 participants with early symptomatic AD receiving oral semaglutide up to 14 mg daily, despite changes in selected cerebrospinal fluid (CSF) biomarkers [96]. Separately, a primary congress report detected semaglutide in CSF after 12 weeks of subcutaneous treatment [12]. This finding supports central exposure but does not demonstrate receptor engagement in brain tissue. These results distinguish biological effects from clinical benefit and do not establish prevention of diabetes-associated cognitive decline.

### 8.4. Comparative Interpretation of Randomised and Observational Evidence

The comparison depends on the endpoint. Both classes have established systemic benefits; for SGLT2 inhibitors, the evidence relevant to this framework is strongest for cardiorenal outcomes, while cognitive findings are mainly observational. GLP-1 RAs combine substantial metabolic and weight-loss effects with small cognitive and neuroimaging studies, observational dementia signals, exploratory randomised findings, and negative phase 3 results in early symptomatic AD [32,33,34,86,87,88,89,90,91,92,93,94,96,97]. The Glycemia Reduction Approaches in Diabetes: A Comparative Effectiveness Study (GRADE) trial found no significant between-group differences in cognitive change among four treatments added to metformin: insulin glargine U-100, glimepiride, liraglutide, and sitagliptin. The trial did not include an SGLT2 inhibitor [77]. The current evidence supports head-to-head, cognition-focused trials with active comparators; it does not justify prescribing either class specifically to prevent dementia. Trials of weight-lowering treatment should separate the effects of reduced excess adiposity from loss of lean mass. In older or frail participants, strength, function, nutrition, and body composition also require monitoring [97]. Table 3 compares the evidence and its limits across the two drug classes.

## 9. Discussion

The reviewed evidence supports evaluating the framework as a research architecture rather than regarding it as a validated disease model. Its added value depends on whether temporal ordering improves interpretation and prediction beyond simpler alternatives. This discussion therefore compares the proposal with existing models, defines its present clinical boundaries, and states its principal limitations.

### 9.1. Relation to Existing Models and Frameworks

Existing models were designed for different purposes. Biomarker-focused T2DM reviews map candidate molecular, vascular, imaging, and neurodegenerative markers but do not assign them to prespecified within-person temporal tests [3,4]. AD criteria define disease biology [98]; neurovascular models emphasise BBB dysfunction, clearance, and hypoperfusion [99]; life-course models organise modifiable risk across the lifespan [58]; and event-based modelling estimates an abnormality sequence from cross-sectional distributions [100]. Unlike biomarker catalogues or event-based staging, this conceptual, hypothesis-generating framework requires upstream change to precede downstream change and tests that sequence against reverse and non-adjacent paths. It adds a longitudinal testing structure rather than a new biomarker or pathway; it is not a validated clinical model. It requires repeated measurement of diabetes-related exposure, molecular injury, structure and co-pathology, cognition, and function; comparison of forward, reverse, and non-adjacent paths; testing against simpler models; and prespecification of modifiers. Table 4 summarises these operational differences.

The framework’s contribution is methodological rather than biological: it introduces no new biomarkers or pathways. Its incremental value lies in four prespecified commitments: assigning measurements to temporal levels, testing forward adjacent-level coupling against reverse and non-adjacent alternatives, comparing out-of-sample prediction with clinical-risk and unordered biomarker models, and declaring modifiers and refutation criteria before analysis. Thus, it predicts order and lagged relationships, not merely change over time, and specifies when a tested link or a claim of incremental predictive utility should be rejected.

### 9.2. Clinical Implications and Current Boundaries

The framework changes study design more than routine care. NfL, GFAP, APOE genotyping, and MRI are not validated for universal screening in T2DM and remain question-specific research or specialist tools [43,44,50,51]. Clinical action should begin with deteriorating self-management, severe hypoglycaemia, medication errors, or functional change. An abnormal screen requires evaluation of education, language, mood, sleep, sensory impairment, delirium, medication burden, and acute metabolic disturbance rather than automatic attribution to diabetes. Once impairment is established, priorities are safety, treatment simplification, lower hypoglycaemia risk, caregiver support, and cardiorenal and vascular protection.

### 9.3. Limitations

The framework simplifies bidirectional metabolic, vascular, inflammatory, adiposity, muscle, and neurodegenerative processes, and its components rest on unequal evidence. No multimodal cohort or trial has tested the full sequence, proposed molecular intervals are unvalidated, and the worked 10% prediction margin is an illustrative example rather than a universal validity criterion. This narrative review used one principal database, no duplicate screening, and no formal risk-of-bias tool. Accordingly, the framework should guide falsifiable longitudinal research, not classify patients, define a diabetes-specific encephalopathy, or select glucose-lowering treatment. A failed predictive comparison may reflect the selected assays, cadence, or outcome rather than absence of the proposed biology; conversely, improved prediction does not establish mediation or causality.

## 10. Conclusions

Cognitive decline in T2DM reflects combinations of metabolic and vascular exposure, adiposity and muscle health, molecular injury, structural vulnerability, co-pathology, reserve, and function. This conceptual, hypothesis-generating framework assigns measurements to temporal levels, tests forward against reverse paths and simpler models, and permits separate rejection of temporal ordering and incremental value. SGLT2 inhibitors and GLP-1 receptor agonists are complementary systemic probes, but neither has established efficacy in preventing cognitive decline. An illustrative 36-month cohort of 300–500 participants, with final size determined by simulation, could evaluate the framework’s incremental predictive value; intervention studies would still be required to establish causality. Repeated exclusion of a prespecified meaningful forward effect rejects the tested temporal link and lag; failure to meet the held-out prediction threshold rejects incremental predictive utility, without disproving the component biology.

## 11. Future Directions

### 11.1. Worked Longitudinal Validation Design

The principal evidence gap is whether the proposed order exists within individuals and improves prediction. In a worked validation example, a study-specific prespecified improvement in held-out root-mean-square error (RMSE), illustrated here as at least 10%, could be used as a threshold for practical incremental value relative to (i) a clinical-risk model containing age, sex, education, baseline cognition, diabetes duration, and vascular and kidney disease, and (ii) an unordered contemporaneous biomarker model. This illustrative margin is not an observed effect estimate or a universal requirement; it must be defined before model fitting and justified for the intended outcome. In an actual study, the threshold should be justified using the minimally important predictive improvement, measurement reliability, expected optimism, and intended clinical use; calibration, uncertainty intervals, and decision-curve or net-benefit analyses should also be considered.

A minimal full validation cohort would include approximately 300–500 participants, with the final sample determined by simulation using expected attrition, measurement reliability, and the smallest target lagged effect. This order of magnitude permits internal train/test separation and a limited number of prespecified interactions; it is not a universal power calculation. In this illustrative design, exposure and blood injury measures could be obtained at baseline, 3–6, 12, 24, and 36 months; cognition and function at baseline, 12, 24, and 36 months; and harmonised MRI at baseline and 24 or 36 months. Measures should include CGM/HbA1c, blood pressure, eGFR/UACR, waist or visceral adiposity, activity and frailty, a focused injury panel, MRI, an executive/processing-speed composite, memory, and instrumental function.

**Figure 2:**
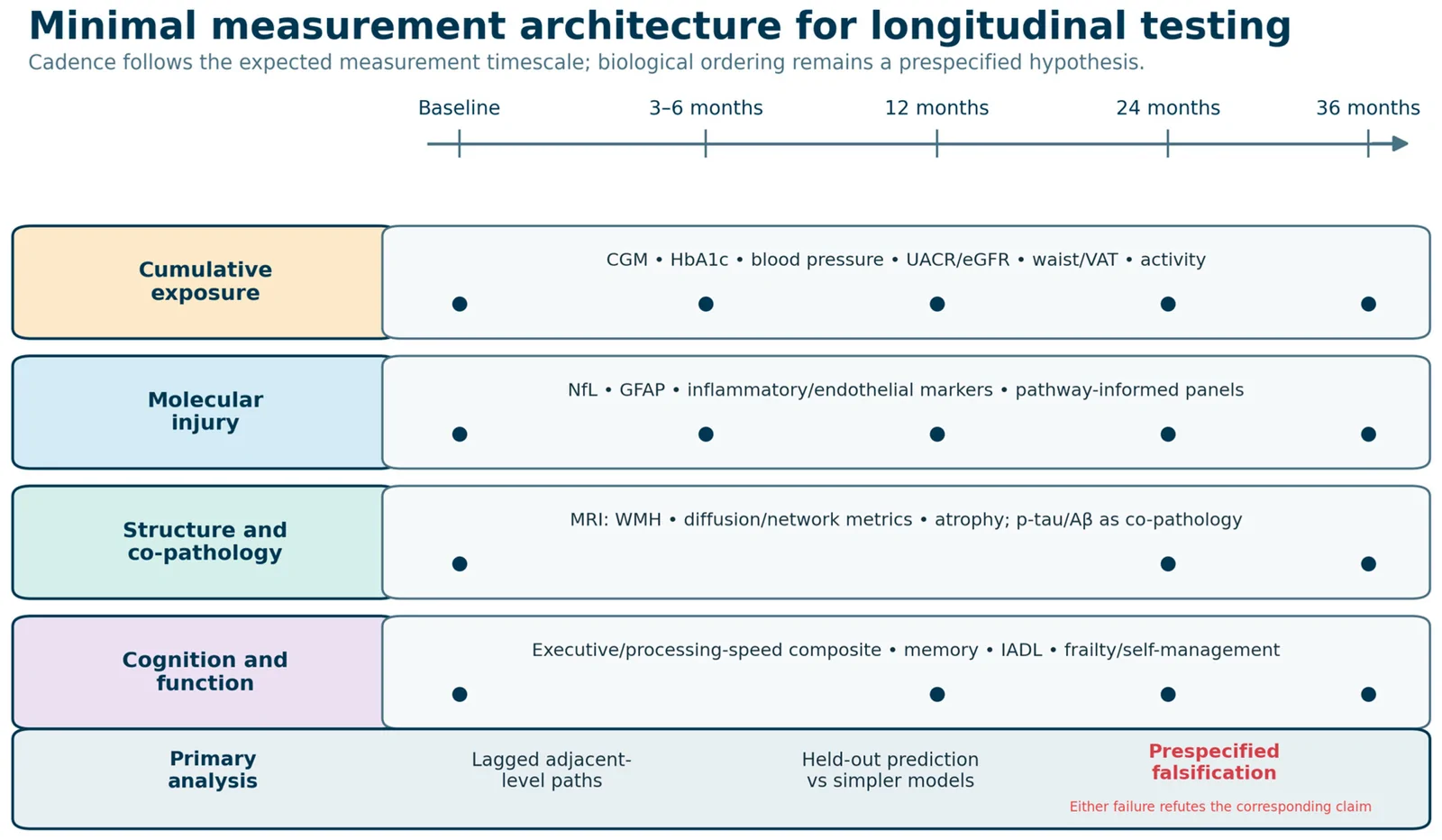
Minimal measurement architecture for longitudinal testing. Exposure and injury measures are repeated at shorter intervals, whereas structural, cognitive, and functional outcomes follow slower expected timescales. An illustrative schedule with assessments at baseline, 3–6, 12, 24, and 36 months permits lagged adjacent-level analyses and comparison with simpler models; the cadence is illustrative, not a validated optimum. Temporal and predictive claims are evaluated separately as defined in Section 11.2; direct pathways do not automatically invalidate adjacent links. Dots mark the planned assessment time points; the coloured left-hand blocks distinguish the exposure, molecular injury, structural/co-pathology, and cognitive/functional levels, while the final row indicates the planned analysis. Abbreviations: Aβ, amyloid-β; CGM, continuous glucose monitoring; eGFR, estimated glomerular filtration rate; GFAP, glial fibrillary acidic protein; HbA1c, glycated haemoglobin; IADL, instrumental activities of daily living; MRI, magnetic resonance imaging; NfL, neurofilament light chain; p-tau, phosphorylated tau; UACR, urinary albumin-to-creatinine ratio; VAT, visceral adipose tissue; WMH, white-matter hyperintensities.

Mixed-effects or latent-change models should separate baseline burden from change, allow nonlinear trajectories, and prespecify practice effects and treatment changes. Cross-lagged or related longitudinal models should compare forward adjacent-level, reverse, and non-adjacent paths while separating stable between-person differences from within-person change [101]. Direct exposure-to-outcome paths should be retained where biologically plausible. Before analysis, each primary link should have a defined lag, expected direction, smallest meaningful effect, and precision requirement. Repeated estimates that exclude the prespecified meaningful forward effect challenge that link; imprecise estimates remain inconclusive. Mediation requires exposure before mediator and mediator before outcome; causal g-methods may be needed when time-varying confounders are affected by earlier exposure. Model evaluation should use held-out or externally validated calibration and prediction, with sensitivity analyses for dropout, intercurrent illness, assay drift, and missing-not-at-random data. The cadence in Figure 2 and Table 5 is pragmatic rather than validated [102,103].

Randomisation is needed to determine whether modifying an upstream exposure alters downstream brain or cognitive trajectories. Observational studies should otherwise use active comparators, new-user or target-trial designs, time-varying covariates, and negative controls where feasible. Genetic instruments and mediation analyses provide triangulation, not substitutes for randomisation.

### 11.2. Interpretation and Refutation

Discordance can refine a link when an independently measured, prespecified modifier explains the mismatch and the interaction replicates. A specified temporal link and lag are rejected when repeated, adequately powered and reliably measured analyses exclude the prespecified meaningful forward effect, or favour an incompatible reverse ordering. A stronger direct, non-adjacent path does not alone reject an adjacent link because direct and mediated effects can coexist. Failure of one link challenges that part of the proposed sequence, not every component mechanism. Separately, failure to meet the prespecified improvement in held-out prediction rejects incremental predictive value for the selected measurements, population, comparator, and outcome; it does not establish biological falsity. Improved prediction likewise does not prove causality. Measurement reliability, lag adequacy, attrition, multiplicity, and precision criteria should be defined before results are known so that an inconclusive test is distinguished from a disconfirming one.

## Figures and Tables

**Table 1:** Core candidate temporal tests. Intervals are design hypotheses rather than validated biological clocks; additional nodes are reported in Supplementary Table S3. Abbreviations: AGE, advanced glycation end product; BBB, blood–brain barrier; IL, interleukin; NfL, neurofilament light chain; NLRP3, NLR family pyrin domain-containing 3; RAGE, receptor for advanced glycation end products; ROS, reactive oxygen species; UACR, urinary albumin-to-creatinine ratio; WMH, white-matter hyperintensities.

Key Caution	Hypothesised Order/Interval	Candidate Readout	Diabetes-Related Trigger	Molecular Node
Peripheral readouts; kidney function	Exposure (months–years) → pathway signal (months) → structure/cognition	AGEs; soluble RAGE; adhesion molecules	Sustained hyperglycaemia; carbonyl stress	AGE–RAGE/NF-κB
Human brain timing unverified	Metabolic change → inflammation (3–12 months) → glial/axonal change	IL-1β; IL-18; focused panel	Glucose/lipid excess; ROS; adipose inflammation	NLRP3–IL-1β/IL-18
Optional exploratory assay; not cerebral mitophagy	Early cellular response → network/NfL change → atrophy	Peripheral blood cell respiration [31]	Variability; ROS; lipid excess	Mitochondrial quality control

**Table 2:** Selected molecular biomarkers and structural readouts relevant to the trajectory-oriented framework. Maturity refers to current analytical and clinical use in the stated context, not to validation as a diabetes-specific diagnostic or treatment-selection tool. Abbreviations: Aβ, amyloid-β; AD, Alzheimer’s disease; GFAP, glial fibrillary acidic protein; hsCRP, high-sensitivity C-reactive protein; IL-6, interleukin-6; MRI, magnetic resonance imaging; NfL, neurofilament light chain; p-tau, phosphorylated tau; p-tau217, tau phosphorylated at threonine 217; T2DM, type 2 diabetes mellitus; TNF-α, tumour necrosis factor-α.

Maturity and Consolidated Limitation	Role in the Framework	Biomarker or Readout
Analytically mature; nonspecific and influenced by age, kidney function, neuropathy, and co-pathology.	Neuroaxonal injury	NfL
Promising longitudinal marker; affected by sex, age, vascular and amyloid-related processes.	Astrocytic response	GFAP
Clinically advancing in symptomatic specialist populations; not validated for screening asymptomatic T2DM.	AD co-pathology	p-tau/Aβ
Exploratory; systemic source, obesity, infection, CKD, and multiplicity limit inference.	Hypothesis-specific molecular injury	Inflammatory/endothelial panel
Clinically established exposure measures; overlapping metrics require prespecification.	Time-varying glycaemic exposure	CGM/HbA1c
Useful longitudinally with harmonised acquisition; mixed aetiology and scanner drift.	Structural anchor	MRI WMH/diffusion/atrophy
Feasible but method-dependent; BMI alone cannot distinguish fat from lean mass.	Adiposity exposure and physical reserve	VAT/waist and lean mass
Clinically relevant; influenced by comorbidity, mood, sensory status, and reverse causality.	Functional reserve and outcome	Strength, gait, frailty, IADL

**Table 3:** Comparative evidence for SGLT2 inhibitors and GLP-1 receptor agonists as translational probes. Abbreviations: AD, Alzheimer’s disease; fMRI, functional magnetic resonance imaging; fNIRS, functional near-infrared spectroscopy; GLP-1 RA, glucagon-like peptide-1 receptor agonist; RCT, randomised controlled trial; SGLT2, sodium–glucose cotransporter-2; T2DM, type 2 diabetes mellitus.

Framework Interpretation	GLP-1 Receptor Agonists	SGLT2 Inhibitors	Evidence Domain
Upstream probes, not cognitive claims	Strong RCT glycaemic/weight evidence; cardiovascular benefit in relevant populations	Strong RCT cardiorenal evidence	Established systemic effects
Engagement must precede later cognition	Preclinical mechanisms; small memory, fNIRS, and fMRI studies; CSF exposure and biological effects without proven brain receptor engagement	Preclinical neuroprotection; limited human fMRI; no direct brain target engagement	Mechanistic/imaging
Hypothesis-supporting; residual confounding	Lower dementia/AD diagnoses in registers and target-trial emulations	Lower dementia incidence in several active-comparator cohorts	Observational cognition/dementia
Neither class is established for prevention	REWIND exploratory cognitive signal; biomarker substudy overall null; small T2DM studies; ELAD; EVOKE/EVOKE+ showed no clinical slowing despite CSF biomarker changes	No definitive cognition/dementia-prevention RCT	Randomised cognition

**Table 4:** Relationship of the proposed framework to established biomarker, disease-specific, neurovascular, prevention, and event-based models.

Relation to the Present Framework	Strength	Primary Question	Approach
Adds diabetes exposures, mixed pathology, function, and longitudinal coupling.	Disease definition and staging [98]	Is AD biology present, and at what stage?	AD biological criteria
Connects neurovascular mechanisms with metabolic, adiposity, cognitive, and functional levels.	Mechanistic vascular ordering [99]	How does vascular/BBB dysfunction promote neurodegeneration?	Neurovascular models
Specifies measurable injury and structural links within individuals.	Prevention perspective [58]	Which modifiable risks accumulate across life?	Life-course prevention
Requires repeated within-person data and tests forward, reverse, and non-adjacent paths.	Data-driven staging in T2DM [100]	What cross-sectional order best explains abnormality distributions?	Event-based model
Assigns biomarkers to prespecified temporal roles and requires repeated measurement, lagged adjacent-level testing, falsification, and comparison with simpler predictive models.	Broad integration of metabolic, vascular, imaging, and neurodegenerative markers [3,4]	Which biomarkers are associated with brain and cognitive outcomes in T2DM?	Biomarker-focused T2DM reviews
Operational contribution: prespecified temporal order, lagged adjacent-level testing, modifier analysis, falsification, and held-out comparison; no claim of new biological components.	Prespecified timing, modifiers, falsification, and held-out comparison	Does ordered coupling improve prediction?	Trajectory framework

**Table 5:** Core questions for future testing of the trajectory-oriented framework.

Refinement Versus Refutation	Primary Test	Measures and Cadence	Framework Link
Refine only with prespecified kidney/systemic modifier; refute if ordering repeatedly fails.	Forward lagged path versus reverse path	CGM/HbA1c, BP, eGFR/UACR, VAT; focused injury panel at baseline, 3–6, 12, 24, 36 months	Exposure → injury
Refine for co-pathology/assay effects; reject tested link if meaningful forward coupling is repeatedly excluded; direct paths may coexist.	Injury change predicts WMH/diffusion/atrophy change	NfL/GFAP/pathway panel; MRI baseline and 24/36 months	Injury → structure
Refine for reserve, sex/gender, frailty; refute absent reproducible coupling.	Structure change predicts domain-specific and functional change	MRI; cognition and IADL/frailty baseline, 12, 24, 36 months	Structure → cognition/function
Reject tested temporal links under prespecified reliability and precision criteria; reject incremental predictive utility if its threshold is not met.	Forward versus reverse/non-adjacent paths; held-out RMSE versus comparators	Study-specific threshold; worked example: ≥10% lower held-out RMSE	Whole model

## Data Availability

No new data were created or analysed in this study.

## Supplementary Materials

The following supporting information can be downloaded at: https://www.mdpi.com/article/10.3390/ijms27188342/s1.

## Abbreviations

The following abbreviations are used in this manuscript.

Abbreviation	Definition
AD	Alzheimer’s disease
AGE	advanced glycation end product
Akt	protein kinase B
AMPK	AMP-activated protein kinase
APOE	Apolipoprotein E
Aβ	amyloid-β
BBB	blood–brain barrier
BDNF	brain-derived neurotrophic factor
BMI	body mass index
CGM	continuous glucose monitoring
CKD	chronic kidney disease
CSF	cerebrospinal fluid
CSVD	cerebral small-vessel disease
CV	coefficient of variation
DNA	deoxyribonucleic acid
DPP-4	dipeptidyl peptidase-4
DSB	Digit Span Backward
DSST	Digit Symbol Substitution Test
eGFR	estimated glomerular filtration rate
ELAD	Evaluating Liraglutide in Alzheimer’s Disease
eNOS	endothelial nitric oxide synthase
FINGER	Finnish Geriatric Intervention Study to Prevent Cognitive Impairment and Disability
fMRI	functional magnetic resonance imaging
fNIRS	functional near-infrared spectroscopy
GFAP	glial fibrillary acidic protein
GLP-1 RA	glucagon-like peptide-1 receptor agonist
GRADE	Glycemia Reduction Approaches in Diabetes: A Comparative Effectiveness Study
GSK-3β	glycogen synthase kinase-3β
HbA1c	glycated haemoglobin
hsCRP	high-sensitivity C-reactive protein
IADL	instrumental activities of daily living
ICAM-1	intercellular adhesion molecule-1
IL	interleukin
IL-18	interleukin-18
IL-1β	interleukin-1β
IL-6	interleukin-6
IR	insulin receptor
Look AHEAD	Action for Health in Diabetes
MAGE	mean amplitude of glycaemic excursions
MCI	mild cognitive impairment
MMSE	Mini-Mental State Examination
MoCA	Montreal Cognitive Assessment
MRI	magnetic resonance imaging
mTOR	mechanistic target of rapamycin
NADPH	nicotinamide adenine dinucleotide phosphate
NF-κB	nuclear factor kappa B
NfL	neurofilament light chain
NLRP3	NLR family pyrin domain-containing 3
NO	nitric oxide
p-tau	phosphorylated tau
p-tau217	tau phosphorylated at threonine 217
p-tau231	tau phosphorylated at threonine 231
PET	positron emission tomography
PGC-1α	peroxisome proliferator-activated receptor-γ coactivator-1α
PI3K	phosphoinositide 3-kinase
PKC	protein kinase C
PRISMA	Preferred Reporting Items for Systematic Reviews and Meta-Analyses
RAGE	receptor for advanced glycation end products
REWIND	Researching Cardiovascular Events with a Weekly Incretin in Diabetes
RMSE	root-mean-square error
RNA	ribonucleic acid
ROS	reactive oxygen species
SANRA	Scale for the Assessment of Narrative Review Articles
SD	standard deviation
SGLT2	sodium–glucose cotransporter 2
SIRT1	sirtuin 1
SPRINT	Systolic Blood Pressure Intervention Trial
T2DM	type 2 diabetes mellitus
TAR	time above range
TBR	time below range
TIR	time in range
TMT-A	Trail Making Test Part A
TMT-B	Trail Making Test Part B
TNF-α	tumour necrosis factor-α
UACR	urinary albumin-to-creatinine ratio
VAT	visceral adipose tissue
VCAM-1	vascular cell adhesion molecule-1
WMH	white matter hyperintensities

## References

[1] Zhao Y., Wang H., Tang G., Wang L., Tian X., Li R. (2025). Risk Factors for Mild Cognitive Impairment in Type 2 Diabetes: A Systematic Review and Meta-Analysis. Front. Endocrinol..

[2] You Y., Liu Z., Chen Y., Xu Y., Qin J., Guo S., Huang J., Tao J. (2021). The Prevalence of Mild Cognitive Impairment in Type 2 Diabetes Mellitus Patients: A Systematic Review and Meta-Analysis. Acta Diabetol..

[3] Biessels G.J., Nobili F., Teunissen C.E., Simó R., Scheltens P. (2020). Understanding Multifactorial Brain Changes in Type 2 Diabetes: A Biomarker Perspective. Lancet Neurol..

[4] Ehtewish H., Arredouani A., El-Agnaf O. (2022). Diagnostic, Prognostic, and Mechanistic Biomarkers of Diabetes Mellitus-Associated Cognitive Decline. Int. J. Mol. Sci..

[5] van Sloten T.T., Sedaghat S., Carnethon M.R., Launer L.J., Stehouwer C.D.A. (2020). Cerebral Microvascular Complications of Type 2 Diabetes: Stroke, Cognitive Dysfunction, and Depression. Lancet Diabetes Endocrinol..

[6] Grillo C.A., Woodruff J.L., Macht V.A., Reagan L.P. (2019). Insulin Resistance and Hippocampal Dysfunction: Disentangling Peripheral and Brain Causes from Consequences. Exp. Neurol..

[7] Dutta B.J., Singh S., Seksaria S., Das Gupta G., Singh A. (2022). Inside the Diabetic Brain: Insulin Resistance and Molecular Mechanism Associated with Cognitive Impairment and Its Possible Therapeutic Strategies. Pharmacol. Res..

[8] Wątroba M., Grabowska A.D., Szukiewicz D. (2023). Effects of Diabetes Mellitus-Related Dysglycemia on the Functions of Blood–Brain Barrier and the Risk of Dementia. Int. J. Mol. Sci..

[9] Kan W., Qu M., Wang Y., Zhang X., Xu L. (2025). A Review of Type 2 Diabetes Mellitus and Cognitive Impairment. Front. Endocrinol..

[10] Liao X., Zhang Y., Xu J., Yin J., Li S., Dong K., Shi X., Xu W., Ma D., Chen X. (2025). Narrative Review on Cognitive Impairment in Type 2 Diabetes: Global Trends and Diagnostic Approaches. Biomedicines.

[11] Mei J., Li Y., Niu L., Liang R., Tang M., Cai Q., Xu J., Zhang D., Yin X., Liu X. (2024). SGLT2 Inhibitors: A Novel Therapy for Cognitive Impairment via Multifaceted Effects on the Nervous System. Transl. Neurodegener..

[12] Alford S., Johannsen P., Bentsen M., Rausch D.B., Carstensen L., Jeppesen R., Martino G., Jiménez-Mausbach M., Knudsen L. Semaglutide Concentration in Cerebrospinal Fluid from Patients with Early Alzheimer’s Disease After 12 Weeks of Subcutaneous Treatment. Presented at the American Academy of Neurology Annual Meeting.

[13] Baethge C., Goldbeck-Wood S., Mertens S. (2019). SANRA—A Scale for the Quality Assessment of Narrative Review Articles. Res. Integr. Peer Rev..

[14] Barbiellini Amidei C., Fayosse A., Dumurgier J., Machado-Fragua M.D., Tabak A.G., van Sloten T., Kivimäki M., Dugravot A., Sabia S., Singh-Manoux A. (2021). Association Between Age at Diabetes Onset and Subsequent Risk of Dementia. JAMA.

[15] LeRoith D., Biessels G.J., Braithwaite S.S., Casanueva F.F., Draznin B., Halter J.B., Hirsch I.B., McDonnell M.E., Molitch M.E., Murad M.H. (2019). Treatment of Diabetes in Older Adults: An Endocrine Society* Clinical Practice Guideline. J. Clin. Endocrinol. Metab..

[16] Rom S., Heldt N.A., Gajghate S., Seliga A., Reichenbach N.L., Persidsky Y. (2020). Hyperglycemia and Advanced Glycation End Products Disrupt BBB and Promote Occludin and Claudin-5 Protein Secretion on Extracellular Microvesicles. Sci. Rep..

[17] Xie Z., Wang X., Luo X., Yan J., Zhang J., Sun R., Luo A., Li S. (2023). Activated AMPK Mitigates Diabetes-Related Cognitive Dysfunction by Inhibiting Hippocampal Ferroptosis. Biochem. Pharmacol..

[18] Li X., Preckel B., Hermanides J., Hollmann M.W., Zuurbier C.J., Weber N.C. (2022). Amelioration of Endothelial Dysfunction by Sodium Glucose Co-Transporter 2 Inhibitors: Pieces of the Puzzle Explaining Their Cardiovascular Protection. Br. J. Pharmacol..

[19] Chi H., Song M., Zhang J., Zhou J., Liu D. (2023). Relationship between Acute Glucose Variability and Cognitive Decline in Type 2 Diabetes: A Systematic Review and Meta-Analysis. PLoS ONE.

[20] Ding J., Shi Q., Tao Q., Su H., Du Y., Pan T., Zhong X. (2023). Correlation between Long-Term Glycemic Variability and Cognitive Function in Middle-Aged and Elderly Patients with Type 2 Diabetes Mellitus: A Retrospective Study. PeerJ.

[21] Cui X., Abduljalil A., Manor B.D., Peng C.-K., Novak V. (2014). Multi-Scale Glycemic Variability: A Link to Gray Matter Atrophy and Cognitive Decline in Type 2 Diabetes. PLoS ONE.

[22] Xia W., Luo Y., Chen Y.-C., Chen H., Ma J., Yin X. (2020). Glucose Fluctuations Are Linked to Disrupted Brain Functional Architecture and Cognitive Impairment. J. Alzheimer’s Dis..

[23] Wang D.-Q., Wang L., Wei M.-M., Xia X.-S., Tian X.-L., Cui X.-H., Li X. (2020). Relationship Between Type 2 Diabetes and White Matter Hyperintensity: A Systematic Review. Front. Endocrinol..

[24] Qiao L., Tang X., Peng J., Xie Q., Wu M., Tang Z. (2026). Blood and Imaging Biomarkers of Blood-Brain Barrier Disruption in Diabetic Individuals with Cognitive Impairment. Aging Clin. Exp. Res..

[25] Tian Y., Jing G., Ma M., Yin R., Zhang M. (2024). Microglial Activation and Polarization in Type 2 Diabetes-Related Cognitive Impairment: A Focused Review of Pathogenesis. Neurosci. Biobehav. Rev..

[26] Luo Y., Zhu J., Hu Z., Luo W., Du X., Hu H., Peng S. (2024). Progress in the Pathogenesis of Diabetic Encephalopathy: The Key Role of Neuroinflammation. Diabetes Metab. Res. Rev..

[27] Zhu W., Zhang H., Niu T., Liu K., Fareeduddin Mohammed Farooqui H., Sun R., Chen X., Yuan Y., Wang S. (2024). Microglial SCAP Deficiency Protects against Diabetes-Associated Cognitive Impairment through Inhibiting NLRP3 Inflammasome-Mediated Neuroinflammation. Brain Behav. Immun..

[28] Lin L., Wu Y., Chen Z., Huang L., Wang L., Liu L. (2021). Severe Hypoglycemia Contributing to Cognitive Dysfunction in Diabetic Mice Is Associated with Pericyte and Blood–Brain Barrier Dysfunction. Front. Aging Neurosci..

[29] Arslan B., Brum W.S., Pola I., Therriault J., Rahmouni N., Stevenson J., Servaes S., Tan K., Vitali P., Montembeault M. (2025). The Impact of Kidney Function on Alzheimer’s Disease Blood Biomarkers: Implications for Predicting Amyloid-β Positivity. Alzheimer’s Res. Ther..

[30] Wang D., Liu J., Zhong L., Li S., Zhou L., Zhang Q., Li M., Xiao X. (2022). The Effect of Sodium-Glucose Cotransporter 2 Inhibitors on Biomarkers of Inflammation: A Systematic Review and Meta-Analysis of Randomized Controlled Trials. Front. Pharmacol..

[31] Hsiao C.-P., Hoppel C.L. (2018). Analyzing Mitochondrial Function in Human Peripheral Blood Mononuclear Cells. Anal. Biochem..

[32] Pachter D., Klein H., Kamer O., Goldberg Toren D.T., Alufer L., Ebstein Karamani N., Atlas T., Yaary A., Hagbi I., Chassidim Y. (2026). Sustained Visceral Fat Loss Is Associated with Attenuated Brain Atrophy and Improved Cognition in Late Midlife. Nat. Commun..

[33] Sun M., Lu Z., Chen W.-M., Wu S.-Y., Zhang J. (2024). Sarcopenia and Diabetes-Induced Dementia Risk. Brain Commun..

[34] Sha T., Zhang Y., Wei J., Li C., Zeng C., Lei G., Wang Y. (2025). Sarcopenia and Risk of Cognitive Impairment: Cohort and Mendelian Randomization Analyses. JMIR Aging.

[35] Wu K., Huang C., Zheng W., Wu Y., Huang Q., Lin M., Gao R., Qi L., He G., Liu X. (2024). Activation of Mitophagy Improves Cognitive Dysfunction in Diabetic Mice with Recurrent Non-Severe Hypoglycemia. Mol. Cell. Endocrinol..

[36] Ciardullo S., Muraca E., Bianconi E., Cannistraci R., Perra S., Zerbini F., Perseghin G. (2023). Diabetes Mellitus Is Associated with Higher Serum Neurofilament Light Chain Levels in the General US Population. J. Clin. Endocrinol. Metab..

[37] Li L., Li C., Zhu L., Zhu J. (2025). Association between Serum NfL and Cognitive Functioning in Older Adults with Diagnosed Diabetes: A Cross-Sectional Study from NHANES. Medicine.

[38] Thota R.N., Chatterjee P., Pedrini S., Hone E., Ferguson J.J.A., Garg M.L., Martins R.N. (2022). Association of Plasma Neurofilament Light Chain with Glycaemic Control and Insulin Resistance in Middle-Aged Adults. Front. Endocrinol..

[39] Rajan K.B., Aggarwal N.T., McAninch E.A., Weuve J., Barnes L.L., Wilson R.S., De Carli C., Evans D.A. (2020). Remote Blood Biomarkers of Longitudinal Cognitive Outcomes in a Population Study. Ann. Neurol..

[40] Axelsson T., Zetterberg H., Blennow K., Arslan B., Ashton N.J., Axelsson M., Svensson M.K., Saeed A., Guron G. (2025). Plasma Concentrations of Neurofilament Light, p-Tau231 and Glial Fibrillary Acidic Protein Are Elevated in Patients with Chronic Kidney Disease and Correlate with Measured Glomerular Filtration Rate. BMC Nephrol..

[41] Mielke M.M., Evans J.K., Neiberg R.H., Molina-Henry D.P., Marcovina S.M., Johnson K.C., Carmichael O.T., Rapp S.R., Sachs B.C., Ding J. (2025). Alzheimer Disease Blood Biomarkers and Cognition Among Individuals with Diabetes and Overweight or Obesity. JAMA Netw. Open.

[42] Raghavan S., Graff-Radford J., Hofrenning E., Fought A.J., Reid R.I., Kamykowski M.G., Algeciras-Schimnich A., Windham B.G., Knopman D.S., Lowe V.J. (2025). Plasma NfL and GFAP for Predicting VCI and Related Brain Changes in Community and Clinical Cohorts. Alzheimer’s Dement..

[43] Ayala-Guerrero L., García-delaTorre P., Sánchez-García S., Guzmán-Ramos K. (2022). Serum Levels of Glial Fibrillary Acidic Protein Association with Cognitive Impairment and Type 2 Diabetes. Arch. Med. Res..

[44] Gonzales M.M., Vela G., Philip V., Trevino H., LaRoche A., Wang C.-P., Parent D.M., Kautz T., Satizabal C.L., Tanner J. (2023). Demographic and Clinical Characteristics Associated with Serum GFAP Levels in an Ethnically Diverse Cohort. Neurology.

[45] Meng F., Fu J., Zhang L., Guo M., Zhuang P., Yin Q., Zhang Y. (2023). Function and Therapeutic Value of Astrocytes in Diabetic Cognitive Impairment. Neurochem. Int..

[46] Palmqvist S., Warmenhoven N., Anastasi F., Pilotto A., Janelidze S., Tideman P., Stomrud E., Mattsson-Carlgren N., Smith R., Ossenkoppele R. (2025). Plasma Phospho-Tau217 for Alzheimer’s Disease Diagnosis in Primary and Secondary Care Using a Fully Automated Platform. Nat. Med..

[47] Bolton C.J., Khan O.A., Liu D., Pechman K.R., Gifford K.A., Hohman T.J., Blennow K., Zetterberg H., Jefferson A.L. (2025). Combining Plasma P-tau231 and Glial Fibrillary Acidic Protein Produces Higher Discriminative Accuracy for Amyloid Positivity than Other Blood-based Biomarker Combinations. Alzheimer’s Dement..

[48] Palmqvist S., Whitson H.E., Allen L.A., Suarez-Calvet M., Galasko D., Karikari T.K., Okrahvi H.R., Paczynski M., Schindler S.E., Teunissen C.E. (2025). Alzheimer’s Association Clinical Practice Guideline on the Use of Blood-Based Biomarkers in the Diagnostic Workup of Suspected Alzheimer’s Disease within Specialized Care Settings. Alzheimer’s Dement..

[49] Cheng Y., Wei L., Chen Y.-H., Fan Y.-T., Lin Y.-N., Martinez R.M., Goh K.K., Chen Y.-C., Jian H.-Y., Chen C. (2026). A Neuroimaging Functional Connectivity Signature of Emotional Conflict Monitoring Predicting Cognitive Decline in Type 2 Diabetes. Sci. Rep..

[50] Roy B., Choi S.E., Freeby M.J., Kumar R. (2023). Microstructural Brain Tissue Changes Contribute to Cognitive and Mood Deficits in Adults with Type 2 Diabetes Mellitus. Sci. Rep..

[51] Moran C., Beare R., Phan T.G., Bruce D.G., Callisaya M.L., Srikanth V., On behalf of the Alzheimer’s Disease Neuroimaging Initiative (ADNI) (2015). Type 2 Diabetes Mellitus and Biomarkers of Neurodegeneration. Neurology.

[52] Johnson L.A., Torres E.R.S., Impey S., Stevens J.F., Raber J. (2017). Apolipoprotein E4 and Insulin Resistance Interact to Impair Cognition and Alter the Epigenome and Metabolome. Sci. Rep..

[53] Wang Y., Gao Y., Wang Y., Zhang F., Sun F., Wang X., Xie J., Xu Z., Zhang J., Xu H. (2025). ApoE4 Upregulates GSK-3β to Aggravate Alzheimer-Like Pathologies and Cognitive Impairment in Type 2 Diabetic Mice. CNS Neurosci. Ther..

[54] Litkowski E.M., Logue M.W., Zhang R., Charest B.R., Lange E.M., Hokanson J.E., Lynch J.A., Vujkovic M., Phillips L.S., Lange L.A. (2022). A Diabetes Genetic Risk Score Is Associated with All-Cause Dementia and Clinically Diagnosed Vascular Dementia in the Million Veteran Program. Diabetes Care.

[55] Mollon J., Curran J.E., Mathias S.R., Knowles E.E.M., Carlisle P., Fox P.T., Olvera R.L., Göring H.H.H., Rodrigue A., Almasy L. (2020). Neurocognitive Impairment in Type 2 Diabetes: Evidence for Shared Genetic Aetiology. Diabetologia.

[56] Luo M., Sun M., Wang T., Wei J., Ruan X., Chen K., Ou J., Chen Y., Qin J. (2024). Type 2 Diabetes, Glycaemic Traits, Structural Brain Capacity and Cognitive Function: A Mendelian Randomization Analysis. Diabetes Obes. Metab..

[57] Zhao R.R., O’Sullivan A.J., Fiatarone Singh M.A. (2018). Exercise or Physical Activity and Cognitive Function in Adults with Type 2 Diabetes, Insulin Resistance or Impaired Glucose Tolerance: A Systematic Review. Eur. Rev. Aging Phys. Act..

[58] Livingston G., Huntley J., Liu K.Y., Costafreda S.G., Selbæk G., Alladi S., Ames D., Banerjee S., Burns A., Brayne C. (2024). Dementia Prevention, Intervention, and Care: 2024 Report of the Lancet Standing Commission. Lancet.

[59] Stern Y., Arenaza-Urquijo E.M., Bartrés-Faz D., Belleville S., Cantilon M., Chetelat G., Ewers M., Franzmeier N., Kempermann G., Kremen W.S. (2020). Whitepaper: Defining and Investigating Cognitive Reserve, Brain Reserve, and Brain Maintenance. Alzheimer’s Dement..

[60] Palmer Allred N.D., Raffield L.M., Hardy J.C., Hsu F.-C., Divers J., Xu J., Smith S.C., Hugenschmidt C.E., Wagner B.C., Whitlow C.T. (2016). APOE Genotypes Associate with Cognitive Performance but Not Cerebral Structure: Diabetes Heart Study MIND. Diabetes Care.

[61] Lee B.C., Choe Y.M., Suh G.-H., Choi I.-G., Lee J.H., Kim H.S., Hwang J., Yi D., Kim J.W. (2022). A Combination of Midlife Diabetes Mellitus and the Apolipoprotein E Ε4 Allele Increase Risk for Cognitive Decline. Front. Aging Neurosci..

[62] Dore G.A., Elias M.F., Robbins M.A., Elias P.K., Nagy Z. (2009). Presence of the APOE Ε4 Allele Modifies the Relationship between Type 2 Diabetes and Cognitive Performance: The Maine–Syracuse Study. Diabetologia.

[63] Ware E.B., Morataya C., Fu M., Bakulski K.M. (2021). Type 2 Diabetes and Cognitive Status in the Health and Retirement Study: A Mendelian Randomization Approach. Front. Genet..

[64] Fachim H.A., Malipatil N., Siddals K., Donn R., Cortés G.Y., Dalton C.F., Gibson J.M., Heald A.H. (2022). Methylation Status of Exon IV of the Brain-Derived Neurotrophic Factor (BDNF)-Encoding Gene in Patients with Non-Diabetic Hyperglycaemia (NDH) before and after a Lifestyle Intervention. Epigenomes.

[65] Wang J., Wang S., Wang J., Xiao M., Guo Y., Tang Y., Zhang J., Gu J. (2021). Epigenetic Regulation Associated with Sirtuin 1 in Complications of Diabetes Mellitus. Front. Endocrinol..

[66] He W., Chang F., Wang T., Sun B., Chen R., Zhao L. (2024). Serum Brain-Derived Neurotrophic Factor Levels in Type 2 Diabetes Mellitus Patients and Its Association with Cognitive Impairment: A Meta-Analysis. PLoS ONE.

[67] Williamson J.D., Pajewski N.M., Auchus A.P., Bryan R.N., Chelune G., Cheung A.K., Cleveland M.L., Coker L.H., Crowe M.G., SPRINT MIND Investigators for the SPRINT Research Group (2019). Effect of Intensive vs Standard Blood Pressure Control on Probable Dementia: A Randomized Clinical Trial. JAMA.

[68] Ngandu T., Lehtisalo J., Solomon A., Levälahti E., Ahtiluoto S., Antikainen R., Bäckman L., Hänninen T., Jula A., Laatikainen T. (2015). A 2 Year Multidomain Intervention of Diet, Exercise, Cognitive Training, and Vascular Risk Monitoring versus Control to Prevent Cognitive Decline in at-Risk Elderly People (FINGER): A Randomised Controlled Trial. Lancet.

[69] Patel S.M., Kang Y.M., Im K., Neuen B.L., Anker S.D., Bhatt D.L., Butler J., Cherney D.Z.I., Claggett B.L., Fletcher R.A. (2024). Sodium-Glucose Cotransporter-2 Inhibitors and Major Adverse Cardiovascular Outcomes: A SMART-C Collaborative Meta-Analysis. Circulation.

[70] Vaduganathan M., Docherty K.F., Claggett B.L., Jhund P.S., de Boer R.A., Hernandez A.F., Inzucchi S.E., Kosiborod M.N., Lam C.S.P., Martinez F. (2022). SGLT2 Inhibitors in Patients with Heart Failure: A Comprehensive Meta-Analysis of Five Randomised Controlled Trials. Lancet.

[71] Lee H., Park S.-E., Kim E.-Y. (2021). Glycemic Variability Impacted by SGLT2 Inhibitors and GLP 1 Agonists in Patients with Diabetes Mellitus: A Systematic Review and Meta-Analysis. J. Clin. Med..

[72] Kim H.K., Biessels G.J., Yu M.H., Hong N., Lee Y., Lee B.-W., Kang E.S., Cha B.-S., Lee E.J., Lee M. (2024). SGLT2 Inhibitor Use and Risk of Dementia and Parkinson Disease Among Patients with Type 2 Diabetes. Neurology.

[73] Wu C.-Y., Iskander C., Wang C., Xiong L.Y., Shah B.R., Edwards J.D., Kapral M.K., Herrmann N., Lanctôt K.L., Masellis M. (2023). Association of Sodium–Glucose Cotransporter 2 Inhibitors with Time to Dementia: A Population-Based Cohort Study. Diabetes Care.

[74] Tang H., Shao H., Shaaban C.E., Yang K., Brown J., Anton S., Wu Y., Bress A., Donahoo W.T., DeKosky S.T. (2023). Newer Glucose-Lowering Drugs and Risk of Dementia: A Systematic Review and Meta-Analysis of Observational Studies. J. Am. Geriatr. Soc..

[75] Shin A., Koo B.K., Lee J.Y., Kang E.H. (2024). Risk of Dementia after Initiation of Sodium-Glucose Cotransporter-2 Inhibitors versus Dipeptidyl Peptidase-4 Inhibitors in Adults Aged 40–69 Years with Type 2 Diabetes: Population Based Cohort Study. BMJ.

[76] Tang H., Donahoo W.T., DeKosky S.T., Lee Y.A., Kotecha P., Svensson M., Bian J., Guo J. (2025). GLP-1RA and SGLT2i Medications for Type 2 Diabetes and Alzheimer Disease and Related Dementias. JAMA Neurol..

[77] Luchsinger J.A., Rosin S.P., Kazemi E.J., Younes N., Suratt C.E., Fattaleh B.N., Florez H.J., Gonzalez J.S., Hollander P., Hox S.H. (2025). Glucose-Lowering Medications, Glycemia, and Cognitive Outcomes: The GRADE Randomized Clinical Trial. JAMA Intern. Med..

[78] Youn Y.J., Kim S., Jeong H.-J., Ah Y.-M., Yu Y.M. (2024). Sodium-Glucose Cotransporter-2 Inhibitors and Their Potential Role in Dementia Onset and Cognitive Function in Patients with Diabetes Mellitus: A Systematic Review and Meta-Analysis. Front. Neuroendocrinol..

[79] The EMPA-KIDNEY Collaborative Group (2023). Empagliflozin in Patients with Chronic Kidney Disease. N. Engl. J. Med..

[80] Song K., Choi J., Jeong D., Shin D., Ah Y.-M., Lee K.Y., Choi K.H. (2025). Comparative Effects of SGLT2 Inhibitors and Incretin-Based Therapies on Dementia Risk in Type 2 Diabetes: A Systematic Review and Meta-Analysis. Front. Endocrinol..

[81] Park S.J., Kim H.J., Seo M., Byun D.W., Suh K., Yoo M.H., Yang H., Lee I., Kwon S.H., Kim M. (2026). Comparative Risk of the Neurodegenerative Outcomes between Sodium-Glucose Co-Transporter 2 (SGLT2) Inhibitors and Thiazolidinediones in Type 2 Diabetes: A Multicentre Cohort Study Using the Korean Healthcare Database (2014–2025). BMJ Open.

[82] van Ruiten C.C., Veltman D.J., Schrantee A., van Bloemendaal L., Barkhof F., Kramer M.H.H., Nieuwdorp M., IJzerman R.G. (2022). Effects of Dapagliflozin and Combination Therapy with Exenatide on Food-Cue Induced Brain Activation in Patients with Type 2 Diabetes. J. Clin. Endocrinol. Metab..

[83] Watt C., Sanchez-Rangel E., Hwang J.J. (2020). Glycemic Variability and CNS Inflammation: Reviewing the Connection. Nutrients.

[84] Ferrannini E., Baldi S., Frascerra S., Astiarraga B., Heise T., Bizzotto R., Mari A., Pieber T.R., Muscelli E. (2016). Shift to Fatty Substrate Utilization in Response to Sodium–Glucose Cotransporter 2 Inhibition in Subjects Without Diabetes and Patients with Type 2 Diabetes. Diabetes.

[85] Alami M., Zerif E., Khalil A., Hajji N., Ramassamy C., Lacombe G., Laurent B., Cohen A.A., Wikowski J.M., Gris D. (2025). Neuroprotective Effects of SGLT2 Inhibitors Empagliflozin and Dapagliflozin on Aβ1–42-Induced Neurotoxicity and Neuroinflammation in Cellular Models of Alzheimer’s Disease. J. Alzheimer’s Dis..

[86] Vadini F., Simeone P.G., Boccatonda A., Guagnano M.T., Liani R., Tripaldi R., Di Castelnuovo A., Cipollone F., Consoli A., Santilli F. (2020). Liraglutide Improves Memory in Obese Patients with Prediabetes or Early Type 2 Diabetes: A Randomized, Controlled Study. Int. J. Obes..

[87] Li Q., Jia M., Yan Z., Li Q., Sun F., He C., Li Y., Zhou X., Zhang H., Liu X. (2021). Activation of Glucagon-Like Peptide-1 Receptor Ameliorates Cognitive Decline in Type 2 Diabetes Mellitus Through a Metabolism-Independent Pathway. J. Am. Heart Assoc..

[88] Cheng H., Zhang Z., Zhang B., Zhang W., Wang J., Ni W., Miao Y., Liu J., Bi Y. (2022). Enhancement of Impaired Olfactory Neural Activation and Cognitive Capacity by Liraglutide, but Not Dapagliflozin or Acarbose, in Patients with Type 2 Diabetes: A 16-Week Randomized Parallel Comparative Study. Diabetes Care.

[89] Cukierman-Yaffe T., Gerstein H.C., Colhoun H.M., Diaz R., García-Pérez L.-E., Lakshmanan M., Bethel A., Xavier D., Probstfield J., Riddle M.C. (2020). Effect of Dulaglutide on Cognitive Impairment in Type 2 Diabetes: An Exploratory Analysis of the REWIND Trial. Lancet Neurol..

[90] Nørgaard C.H., Friedrich S., Hansen C.T., Gerds T., Ballard C., Møller D.V., Knudsen L.B., Kvist K., Zinman B., Holm E. (2022). Treatment with Glucagon-Like Peptide-1 Receptor Agonists and Incidence of Dementia: Data from Pooled Double-Blind Randomized Controlled Trials and Nationwide Disease and Prescription Registers. Alzheimer’s Dement..

[91] Wilson J.M., Dage J.L., Qian H.-R., Irelan C.L., Crowder H.S., Duffin K.L., Mintun M., Brooks D.A., Gerstein H.C., Bethel M.A. (2026). Dulaglutide and Neurodegeneration Biomarkers: REWIND Post Hoc Analysis. Alzheimer’s Dement..

[92] Cassataro G., Scriffignano S., Geraci G., Augello G., Grasso M.A., D’Ippolito M.E., Puleo M.G., Cadelo M., Renda M., Tuttolomondo A. (2026). Impact of Semaglutide on Cognitive Function in Patients with Type 2 Diabetes and Mild Cognitive Impairment: A 24-Month Observational Study. Intern. Emerg. Med..

[93] Wang W., Wang Q., Qi X., Gurney M., Perry G., Volkow N.D., Davis P.B., Kaelber D.C., Xu R. (2024). Associations of Semaglutide with First-Time Diagnosis of Alzheimer’s Disease in Patients with Type 2 Diabetes: Target Trial Emulation Using Nationwide Real-World Data in the US. Alzheimer’s Dement..

[94] Inoue K., Saliba D., Gotanda H., Moin T., Mangione C.M., Klomhaus A.M., Tsugawa Y. (2025). Glucagon-Like Peptide-1 Receptor Agonists and Incidence of Dementia among Older Adults with Type 2 Diabetes: A Target Trial Emulation. Ann. Intern. Med..

[95] Edison P., Femminella G.D., Ritchie C., Nowell J., Holmes C., Walker Z., Ridha B., Raza S., Livingston N.R., Frangou E. (2026). Liraglutide in Mild to Moderate Alzheimer’s Disease: A Phase 2b Clinical Trial. Nat. Med..

[96] Cummings J.L., Atri A., Sano M., Zetterberg H., Scheltens P., Knop F.K., Johannsen P., Wichmann C.A., Abschneider R.M., Leon T. (2026). Efficacy and Safety of Oral Semaglutide 14 mg (Flexible Dose) in Early-Stage Symptomatic Alzheimer’s Disease (evoke and evoke+): Two Phase 3, Randomised, Placebo-Controlled Trials. Lancet.

[97] Wilding J.P.H., Batterham R.L., Calanna S., Davies M., Van Gaal L.F., Lingvay I., McGowan B.M., Rosenstock J., Tran M.T.D., Wadden T.A. (2021). Once-Weekly Semaglutide in Adults with Overweight or Obesity. N. Engl. J. Med..

[98] Jack C.R., Andrews J.S., Beach T.G., Buracchio T., Dunn B., Graf A., Hansson O., Ho C., Jagust W., McDade E. (2024). Revised Criteria for Diagnosis and Staging of Alzheimer’s Disease: Alzheimer’s Association Workgroup. Alzheimer’s Dement..

[99] Zlokovic B.V. (2011). Neurovascular Pathways to Neurodegeneration in Alzheimer’s Disease and Other Disorders. Nat. Rev. Neurosci..

[100] Ni M.-H., Hu B., Bai X.-Y., Tong Y., Ma Z.-Y., Xie H., Cao X.-Y., Cui Y.-Y., Li S.-N., Dai P. (2025). Temporal Dynamic of Cognitive Decline in Type 2 Diabetes Mellitus Patients: A Multimodal Biomarker Analysis Using Event-Based Modal and Principal Component Analysis. Diabetol. Metab. Syndr..

[101] Hamaker E.L., Kuiper R.M., Grasman R.P.P.P. (2015). A Critique of the Cross-Lagged Panel Model. Psychol. Methods.

[102] Moran C., Beare R., Wang W., Callisaya M., Srikanth V. (2019). for the Alzheimer’s Disease Neuroimaging Initiative (ADNI). Type 2 Diabetes Mellitus, Brain Atrophy, and Cognitive Decline. Neurology.

[103] Lorenzo T., Ngandu T., Lehtisalo J., Antikainen R., Gispert J.D., Kemppainen N., Laatikainen T., Lindström J., Rinne J., Soininen H. (2025). Associations of Prediabetes, Diabetes and Glucose-Related Markers with Cognition and Neuroimaging in a 2-Year Multidomain Lifestyle Randomised Controlled Trial. Diabetes Metab. Res. Rev..

