# Supplementary material for: Trajectory-Oriented Brain Vulnerability Framework for Cognitive Decline in Type 2 Diabetes
Source: Int J Mol Sci. 2026 Sep 19;27(18):8342. doi: 10.3390/ijms27188342 (PMC13607053; doi:10.3390/ijms27188342)
Supplement: Supplementary file 1 [file ijms-27-08342-s001.zip › ijms-4505148-supplementary.pdf]

Supplementary Materials

Trajectory-Oriented Brain Vulnerability Framework for Cognitive Decline in Type 2 Diabetes

Search date: 30 July 2026. Database: PubMed. Coverage: database inception through the search date. The search strings are documented below. No PubMed language filter was applied; English-language eligibility was applied during screening. No global human-studies filter was used because experimental evidence was eligible for the mechanistic questions addressed in the review. Searches were supplemented by backward reference screening and forward citation tracking. Quotation marks, truncation, Boolean operators, parentheses, and field tags are intentional. Targeted contextual and methodological additions made during revision are identified in the relevant passages of the main manuscript; these did not constitute a new exhaustive search.

Supplementary Table S1. PubMed search strategies

**Thematic area**	**Complete PubMed string**	Coverage and use
Core diabetes–cognition	("Diabetes Mellitus, Type 2"[mh] OR "type 2 diabetes"[tiab] OR T2DM[tiab]) AND ("Cognition Disorders"[mh] OR "cognitive decline"[tiab] OR cognit*[tiab] OR "mild cognitive impairment"[tiab] OR MCI[tiab] OR dementia[tiab])	Database inception–30 Jul 2026; title/abstract and reference screening
Clinical phenotype and timing	("Diabetes Mellitus, Type 2"[mh] OR "type 2 diabetes"[tiab] OR T2DM[tiab]) AND (cognit*[tiab] OR "mild cognitive impairment"[tiab] OR MCI[tiab] OR dementia[tiab]) AND (phenotype*[tiab] OR executive[tiab] OR "processing speed"[tiab] OR memory[tiab] OR longitudinal[tiab] OR trajector*[tiab] OR duration[tiab] OR "age at onset"[tiab])	Database inception–30 Jul 2026; title/abstract and reference screening
Molecular and neurovascular mechanisms	("Diabetes Mellitus, Type 2"[mh] OR "type 2 diabetes"[tiab] OR T2DM[tiab]) AND (brain[tiab] OR cognit*[tiab] OR dementia[tiab]) AND ("oxidative stress"[tiab] OR "advanced glycation"[tiab] OR AGE-RAGE[tiab] OR AGEs[tiab] OR RAGE[tiab] OR NF-kappaB[tiab] OR "NF kappa B"[tiab] OR NLRP3[tiab] OR inflammasome[tiab] OR "insulin signaling"[tiab] OR "insulin signalling"[tiab] OR PI3K[tiab] OR GSK-3beta[tiab] OR GSK3B[tiab] OR AMPK[tiab] OR SIRT1[tiab] OR PGC-1alpha[tiab] OR PGC1alpha[tiab] OR mitochondri*[tiab] OR mitophagy[tiab] OR neurovascular[tiab] OR "endothelial dysfunction"[tiab] OR "blood-brain barrier"[tiab] OR BBB[tiab])	Database inception–30 Jul 2026; title/abstract and reference screening
Blood biomarkers	("Diabetes Mellitus, Type 2"[mh] OR "type 2 diabetes"[tiab] OR T2DM[tiab]) AND (cognit*[tiab] OR dementia[tiab] OR brain[tiab]) AND (biomarker*[tiab] OR neurofilament[tiab] OR NfL[tiab] OR GFAP[tiab] OR "glial fibrillary acidic protein"[tiab] OR "phosphorylated tau"[tiab] OR p-tau[tiab] OR p-tau217[tiab] OR p-tau231[tiab] OR ptau217[tiab] OR ptau231[tiab] OR amyloid[tiab] OR "amyloid beta"[tiab] OR BDNF[tiab] OR cytokine*[tiab])	Database inception–30 Jul 2026; title/abstract and reference screening
Neuroimaging and structure	("Diabetes Mellitus, Type 2"[mh] OR "type 2 diabetes"[tiab] OR T2DM[tiab]) AND (cognit*[tiab] OR dementia[tiab] OR brain[tiab] OR neuroimaging[tiab]) AND (MRI[tiab] OR fMRI[tiab] OR DTI[tiab] OR "magnetic resonance imaging"[tiab] OR neuroimaging[tiab] OR "white matter hyperintensities"[tiab] OR WMH[tiab] OR diffusion[tiab] OR atrophy[tiab] OR connectivity[tiab] OR perfusion[tiab] OR "blood-brain barrier"[tiab] OR BBB[tiab])	Database inception–30 Jul 2026; title/abstract and reference screening
Genetics, epigenetics, reserve, sex/gender	("Diabetes Mellitus, Type 2"[mh] OR "type 2 diabetes"[tiab] OR T2DM[tiab]) AND (cognit*[tiab] OR dementia[tiab] OR brain[tiab]) AND (APOE[tiab] OR genetic*[tiab] OR polygenic[tiab] OR epigenetic*[tiab] OR methylation[tiab] OR transcript*[tiab] OR "cognitive reserve"[tiab] OR sex[tiab] OR gender[tiab])	Database inception–30 Jul 2026; title/abstract and reference screening
Behaviour, activity, sleep, mood, and frailty	("Diabetes Mellitus, Type 2"[mh] OR "type 2 diabetes"[tiab] OR T2DM[tiab]) AND (cognit*[tiab] OR "mild cognitive impairment"[tiab] OR MCI[tiab] OR dementia[tiab]) AND ("physical activity"[tiab] OR exercise[tiab] OR sedentary[tiab] OR sleep[tiab] OR depression[tiab] OR stress[tiab] OR frailty[tiab] OR "self-management"[tiab] OR "social support"[tiab] OR hearing[tiab] OR vision[tiab])	Database inception–30 Jul 2026; title/abstract and reference screening

Thematic area	Complete PubMed string	Coverage and use
Adiposity, body composition, and skeletal muscle	("Diabetes Mellitus, Type 2"[mh] OR "type 2 diabetes"[tiab] OR T2DM[tiab] OR obesity[tiab]) AND (cognit*[tiab] OR dementia[tiab] OR brain[tiab]) AND ("visceral adiposity"[tiab] OR "visceral fat"[tiab] OR obesity[tiab] OR metaflammation[tiab] OR "body composition"[tiab] OR "lean mass"[tiab] OR muscle[tiab] OR sarcopenia[tiab] OR frailty[tiab] OR myokine*[tiab] OR adipokine*[tiab])	Database inception–30 Jul 2026; title/abstract and reference screening
SGLT2 inhibitors	("Diabetes Mellitus, Type 2"[mh] OR "type 2 diabetes"[tiab] OR T2DM[tiab]) AND (cognit*[tiab] OR dementia[tiab] OR Alzheimer*[tiab] OR brain[tiab] OR neuroprotect*[tiab]) AND ("Sodium-Glucose Transporter 2 Inhibitors"[mh] OR SGLT2[tiab] OR SGLT2i[tiab] OR gliflozin*[tiab] OR empagliflozin[tiab] OR dapagliflozin[tiab] OR canagliflozin[tiab] OR ertugliflozin[tiab] OR sotagliflozin[tiab])	Database inception–30 Jul 2026; title/abstract and reference screening
GLP-1 receptor agonists	(("Diabetes Mellitus, Type 2"[mh] OR "type 2 diabetes"[tiab] OR T2DM[tiab]) OR (Alzheimer*[tiab] OR dementia[tiab])) AND (cognit*[tiab] OR dementia[tiab] OR Alzheimer*[tiab] OR brain[tiab] OR neuroimaging[tiab] OR neuroprotect*[tiab]) AND ("Glucagon-Like Peptide-1 Receptor Agonists"[mh] OR "GLP-1 receptor agonist"[tiab] OR GLP-1RA[tiab] OR GLP1RA[tiab] OR liraglutide[tiab] OR semaglutide[tiab] OR dulaglutide[tiab] OR exenatide[tiab] OR lixisenatide[tiab] OR albiglutide[tiab])	Database inception–30 Jul 2026; title/abstract and reference screening
Competing frameworks and validation methods	(("type 2 diabetes"[tiab] OR T2DM[tiab]) AND (cognit*[tiab] OR dementia[tiab]) AND (framework[tiab] OR model*[tiab] OR "event-based"[tiab] OR trajector*[tiab])) OR ((biomarker*[tiab] OR neurovascular[tiab] OR "life course"[tiab]) AND (cognit*[tiab] OR dementia[tiab]) AND (longitudinal[tiab] OR framework[tiab] OR model*[tiab]))	Database inception–30 Jul 2026; title/abstract and reference screening

Eligibility and evidence-selection rules

Domain	Evidence-selection rule
Population	Adults with T2DM; mixed populations only when diabetes-specific data were separately interpretable. AD-only trials were contextual and not extrapolated to diabetes-associated prevention.
Eligible evidence	Peer-reviewed human studies reporting cognitive, functional, biomarker, imaging, or treatment outcomes relevant to a framework link. Experimental studies were included only to clarify mechanisms lacking direct human-brain evidence.
Exclusions	Type 1 or gestational diabetes-only studies; duplicate reports; no cognitive, brain, or prespecified mechanistic relevance; non-peer-reviewed reports except consensus/reporting guidance and the explicitly identified primary congress report on CSF semaglutide exposure (main-text reference 101), used only as contextual evidence, not cognitive efficacy.
Prioritisation	Systematic reviews/meta-analyses for breadth; randomised trials for treatment effects; prospective cohorts for temporal associations; cross-sectional studies for hypothesis generation; and experimental work for biological plausibility. Within each domain, priority also reflected direct relevance to the framework link under consideration, methodological design, sample size and population relevance, longitudinality, and ability to inform temporal ordering or clinical interpretation.
Foundational publication	A publication establishing a definition, biological construct, measurement concept, or disease model on which subsequent evidence depended.
Contextual publication	Adjacent evidence informing interpretive boundaries, target engagement, or generalisability without directly studying T2DM-associated cognitive decline.
Conflicting evidence	Retained and weighted by design, directness, precision, consistency, and susceptibility to confounding; not resolved by vote counting.
Citation tracking	Backward screening of included and model-defining publications plus forward citation tracking for major trials, frameworks, and rapidly evolving therapeutic evidence; selection remained subject to the same relevance and evidence-hierarchy rules.

Supplementary Table S2. SANRA self-assessment

SANRA item	Assessment	Location / rationale
1. Justification of importance	Addressed	Introduction defines clinical and scientific relevance of heterogeneous cognitive decline in T2DM.
2. Statement of aims	Addressed	Introduction states the aim of an operational longitudinal exposure–injury–structure–function framework.
3. Description of literature search	Addressed	Sections 2.1–2.3 and Supplementary Table S1 report source, coverage, exact search strings, eligibility, prioritisation, conflict handling, and citation tracking.
4. Referencing	Addressed	Key claims and competing interpretations are referenced; evidence levels are distinguished.
5. Scientific reasoning	Addressed with limitations	Alternative explanations, confounding, temporal requirements, and separate criteria for rejecting a tested temporal link and incremental predictive utility are discussed. Direct paths may coexist with adjacent links.

SANRA item	Assessment	Location / rationale
6. Presentation of endpoint data	Not a quantitative synthesis	No meta-analysis was conducted; where quantitative trial results are central, direction and limitations are reported. The manuscript does not claim systematic effect estimation.

This self-assessment supports transparent narrative-review reporting but is not a substitute for systematic-review methods, duplicate screening, formal risk-of-bias assessment, or PRISMA reporting.

Supplementary Table S3. Additional molecular nodes and candidate temporal tests

These exploratory nodes complement the three core pathways retained in main-text Table 1. The proposed intervals are pragmatic hypotheses for longitudinal study design, not validated human biological clocks. An optional peripheral blood cell respiration assay is described in Section 4.6 and main-text Table 1; it is not a measure of cerebral mitophagy. Temporal and predictive tests follow the distinct criteria in Section 11.2.

Molecular node	Diabetes-related trigger	Candidate readout	Hypothesised order/interval	Key caution
IR–PI3K–Akt–GSK-3β	Insulin resistance	Insulin-resistance and signalling panels	Early signalling change → synaptic/network change → later cognition	Peripheral insulin resistance does not measure brain signalling.
AMPK–SIRT1–PGC-1α	Energetic stress and nutrient excess	Targeted transcript, protein, or metabolomic signatures	Early energy-sensing response → mitochondrial/network change	Predominantly experimental; tissue and cell specificity limit inference.
Endothelium / BBB	Blood pressure, dysglycaemia, CKD, and visceral adiposity	UACR, ICAM-1/VCAM-1, perfusion or BBB imaging	Systemic burden → endothelial change → WMH/diffusion change	Shared vascular and kidney determinants complicate attribution.
Neuroglial / axonal injury	Convergent vascular, metabolic, and co-pathological injury	GFAP and NfL	Injury-marker change over months → structure/cognition over 12–36 months	Sensitive but nonspecific; age, kidney function, and co-pathology confound interpretation.
Adipose–muscle signalling	Visceral adiposity, inactivity, and sarcopenia	VAT/waist, lean mass, strength, and adipokine/myokine panels	Body-composition change → metabolic/inflammatory change → reserve/function	Bidirectionality and treatment-related lean-mass loss require prespecified assessment.
